# Tumor quiescence: elevating SOX2 in diverse tumor cell types downregulates a broad spectrum of the cell cycle machinery and inhibits tumor growth

**DOI:** 10.1186/s12885-020-07370-7

**Published:** 2020-10-01

**Authors:** Ethan P. Metz, Erin L. Wuebben, Phillip J. Wilder, Jesse L. Cox, Kaustubh Datta, Donald Coulter, Angie Rizzino

**Affiliations:** 1 Eppley Institute for Research in Cancer and Allied Diseases Fred & Pamela Buffett Cancer Center, University of Nebraska Medical Center, Omaha, NE 68198-6805 USA; 2 Department of Pathology and Microbiology, University of Nebraska Medical Center Fred & Pamela Buffett Cancer Center, Omaha, NE 68198-6805 USA; 3 Department of Biochemistry and Molecular Biology Fred & Pamela Buffett Cancer Center, University of Nebraska Medical Center, Omaha, NE 68198-6805 USA; 4 Department of Pediatrics, Fred & Pamela Buffett Cancer Center, University of Nebraska Medical Center, Omaha, NE 68198-6805 USA

**Keywords:** SOX2, Prostate cancer, Pancreatic cancer, Medulloblastoma, Neuroblastoma

## Abstract

**Background:**

Quiescent tumor cells pose a major clinical challenge due to their ability to resist conventional chemotherapies and to drive tumor recurrence. Understanding the molecular mechanisms that promote quiescence of tumor cells could help identify therapies to eliminate these cells. Significantly, recent studies have determined that the function of SOX2 in cancer cells is highly dose dependent. Specifically, SOX2 levels in tumor cells are optimized to promote tumor growth: knocking down or elevating SOX2 inhibits proliferation. Furthermore, recent studies have shown that quiescent tumor cells express higher levels of SOX2 compared to adjacent proliferating cells. Currently, the mechanisms through which elevated levels of SOX2 restrict tumor cell proliferation have not been characterized.

**Methods:**

To understand how elevated levels of SOX2 restrict the proliferation of tumor cells, we engineered diverse types of tumor cells for inducible overexpression of SOX2. Using these cells, we examined the effects of elevating SOX2 on their proliferation, both in vitro and in vivo. In addition, we examined how elevating SOX2 influences their expression of cyclins, cyclin-dependent kinases (CDKs), and p27^Kip1^.

**Results:**

Elevating SOX2 in diverse tumor cell types led to growth inhibition in vitro. Significantly, elevating SOX2 in vivo in pancreatic ductal adenocarcinoma, medulloblastoma, and prostate cancer cells induced a reversible state of tumor growth arrest. In all three tumor types, elevation of SOX2 in vivo quickly halted tumor growth. Remarkably, tumor growth resumed rapidly when SOX2 returned to endogenous levels. We also determined that elevation of SOX2 in six tumor cell lines decreased the levels of cyclins and CDKs that control each phase of the cell cycle, while upregulating p27^Kip1^.

**Conclusions:**

Our findings indicate that elevating SOX2 above endogenous levels in a diverse set of tumor cell types leads to growth inhibition both in vitro and in vivo. Moreover, our findings indicate that SOX2 can function as a master regulator by controlling the expression of a broad spectrum of cell cycle machinery. Importantly, our SOX2-inducible tumor studies provide a novel model system for investigating the molecular mechanisms by which elevated levels of SOX2 restrict cell proliferation and tumor growth.

## Background

The stem cell transcription factor SOX2 plays prominent roles during mammalian development, cellular reprogramming, and cancer progression. Early studies established that SOX2 is essential for maintaining the self-renewal and pluripotency of embryonic stem cells [1]. Subsequent studies demonstrated that small increases in SOX2 in embryonic stem cells disrupts their self-renewal and rapidly induces their differentiation into multiple cell types [2]. Interest in SOX2 increased dramatically with the discovery that SOX2 along with Oct4, Klf4, and c-Myc are sufficient to reprogram somatic cells to a pluripotent stem cell state [3]. Consistent with the essential role of SOX2 in pluripotent stem cells, SOX2 null mouse embryos fail to develop past the peri-implantation stage [4].

SOX2 is also required later in development and its dosage is critical [5]. The importance of SOX2 levels was first observed in the developing eye and central nervous system where SOX2 is associated with the maintenance of progenitor cell populations. SOX2 loss of function mutations cause abnormalities in these tissues due to depletion of progenitor cells [6, 7]. Significantly, the functions of SOX2 are highly dependent on its dosage and either elevating or knocking down SOX2 impairs major cell fate decisions. SOX2 dosage also influences the proliferation of normal cells [5]. In many developing tissues, high levels of SOX2 have been linked directly to reduced cell proliferation. In these tissues, a causative relationship between high levels of SOX2 and decreased proliferation was established by knocking down and overexpressing SOX2 in SOX2^high^ and SOX2^low^ fetal cells, respectively [8, 9].

In addition to its critical roles during development, SOX2 has been implicated in over 20 different human cancers [10]. In most of these cancers, SOX2 expression is associated with their respective tumor-initiating/cancer stem cell populations and thus, disease progression and negative outcomes. Similar to the effects of SOX2 during development, elevated levels of SOX2 are associated with quiescent tumor-initiating cells [5]. In a mouse model of sonic hedgehog subgroup medulloblastoma, elevated SOX2 expression is observed in a minor, quiescent subpopulation within the tumor that is able to resist chemotherapy and drive tumor recurrence [11]. Similarly, elevated levels of endogenous SOX2 in colorectal tumor cells are associated with slower proliferation, but higher tumor-initiating capacity, than the SOX2^low^ population of colorectal tumor cells [12]. High endogenous SOX2 expression has also been observed in disseminated human lung tumor cells that can remain dormant for months, while retaining tumor-initiating capacity [13]. Consistent with these studies, reports from our laboratory have shown that elevating SOX2 in several tumor cell lines leads to growth inhibition [14–16]. Furthermore, both inducible overexpression and inducible knockdown of SOX2 in pancreatic ductal adenocarcinoma (PDAC) cells lead to a significant reduction in tumor growth, indicating that SOX2 levels in these cells are optimized to maximize tumor growth [15]. Collectively, our work and that of others supports the hypothesis that elevated levels of SOX2 limit the proliferation of several types of tumor cells. This is of particular interest with respect to tumor recurrence. Quiescent tumor-initiating cells pose a major clinical challenge, because these cells are largely resistant to therapies that target proliferating cells. Gaining insights into the molecular mechanisms by which elevated SOX2 arrests the growth of tumor cells could identify therapeutic targets for the eradication of quiescent tumor-initiating cells, thus helping to address a major unmet need.

To further our understanding of how elevated SOX2 influences the growth of tumor cells, the studies reported here set out to address two questions. First, how does elevating SOX2 in different tumor cell types influence tumor growth? Second, how does elevating SOX2 inhibit the growth of tumor cells; specifically, how does elevation of SOX2 influence the expression of the cell cycle machinery?

## Methods

### Cell culture

T3M4, HPAF-II, LNCaP and their counterparts engineered for inducible overexpression of SOX2, i-SOX2-T3M4, i-SOX2-HPAF-II, i-SOX2-LNCaP have been described previously [15, 16]. ONS76 cells [17] were obtained from Sutapa Ray (UNMC, Omaha, NE). BE(2)C, DU145, and PC3 cells were obtained from American Type Culture Collection (Manassas, VA). Cells were engineered for Dox-inducible SOX2 expression, as described previously [14–16]. All parental cells, as well as i-SOX2-derivatives, were cultured in Dulbecco’s Modified Eagle Medium supplemented with 10% fetal bovine serum. Palbociclib was obtained from Selleckchem (Houston, TX) and suspended in DMSO. Doxycycline (Dox, Clontech, Mountain View, CA) was suspended in phosphate buffered saline (PBS). To induce SOX2 overexpression, Dox was added to the culture medium at the concentrations and the times indicated. MTT assays were used to assess relative cell growth in triplicate samples, as described previously [14–16]. To complement the results obtained by MTT assays, SOX2-inducible cells were cultured in the presence or absence of Dox for 4 days after which EdU was added to the culture medium for 2 h. Samples were then prepared using a Click-iT EdU flow cytometry kit (ThermoFisher, Rockford, IL) according to the manufacturer’s protocol. Flow cytometry analysis was performed by the UNMC Flow Cytometry core facility. Statistical significance was determined using a two-tailed students t-test with a significance threshold of *p* = 0.05. Cell lines not obtained from the American Type Culture Collection were verified by genetic analysis, which was performed by the Molecular Diagnostics Laboratory at this medical center. Cells were also tested for the presence of mycoplasma and found to be negative.

### In vivo tumor studies

Animal studies were approved by the University of Nebraska Medical Center IACUC Committee and were carried out in accordance with the relevant guidelines and regulations. Female NCr-nu/nu mice (4 weeks old) were purchased from Charles River (Wilmington, MA). All animal protocols were approved by the UNMC Institutional Animal Care and Use Committee. For i-SOX2-T3M4 and i-SOX2-HPAF-II 2.5 × 10^5^ cells were resuspended in sterile PBS prior to subcutaneous transplantation. For i-SOX2-DU145, i-SOX2-PC3, and i-SOX2-ONS76 1.5 × 10^6^ cells were resuspended in 3:1 sterile PBS:Matrigel (Corning, Corning, NY) and transplanted subcutaneously. In each case, the cells were injected into the right hind flank. After injection, tumor growth was monitored daily. When palpable tumors formed, tumor-bearing mice were randomly sorted into size-matched control and Dox-treated groups. Dox was administered at a dose of 2 mg/ml (Sigma-Aldrich, St. Louis, MO) in drinking water containing 5% sucrose as indicated. Control mice were given 5% sucrose drinking water. Tumor volumes were calculated based on digital caliper measurements at the indicated times, as described previously [15]. At the completion of the study, mice were euthanized by CO_2_ asphyxiation for 5 min followed by cervical dislocation and tumors were excised for immunohistochemical analysis. Tumor growth curves were plotted using Microsoft Excel. Analysis of formalin-fixed, paraffin-embedded tumors was performed, as previously described [15]. Statistical significance was determined using a two-tailed students t-test with a significance threshold of *p* = 0.05.

### Western blotting

Whole cell protein was extracted using RIPA buffer (ThermoFisher, Rockford, IL). RIPA buffer was supplemented with protease and phosphatase inhibitors and western blot analysis was performed, as described previously [18, 19]. The following antibodies from Cell Signaling Technology (Danvers MA) were used for western blot analyses: SOX2 (#3579,1:1000), cyclin D1 (#2978, 1:1000), cyclin E1 (#4129, 1:1000), cyclin A2 (#4656, 1:1000), cyclin B1 (#4138, 1:1000), CDK4 (#12790, 1:1000), CDK2 (#2546, 1:1000), CDK1 (#9116, 1:1000), p27^Kip1^ (#3686, 1:1000), HDAC1 (#34586, 1:1000), and β-Tubulin (#2146, 1:1000). HDAC1 and β-Tubulin antibodies were used as loading controls. Rabbit primary antibodies were detected with an anti-rabbit-IgG-AP secondary antibody (A3687, Sigma-Aldrich, 1:5000), as described previously [18, 19]. Mouse primary antibodies were detected with an anti-mouse-IgG-AP secondary antibody (A4312, Sigma-Aldrich, 1:5000). Changes in cell cycle-related proteins were confirmed for each cell line by repeating each western blot analysis at least twice.

### Cell cycle analysis

i-SOX2 cells were seeded at subconfluent densities and cultured in the presence or absence of Dox for 4 days. Cells were then prepared for cell cycle analysis by the Telford method, as described previously [14]. Floating cells were harvested and included in the analysis. Flow cytometry analysis was performed by the UNMC Flow Cytometry core facility. To complement the cell cycle results obtained using the Telford method, cells were transduced with the FastFUCCI lentiviral cell cycle indicator [20]. After subculturing the transduced cells, they were cultured in the presence or absence of the indicated doses of Dox for 4 days, then harvested and analyzed for fluorescent signal by the UNMC Flow Cytometry core facility.

### Immunohistochemistry (IHC)

At the conclusion of the tumor growth studies, mice were euthanized and tumors were excised and processed for IHC. Formalin-fixed tumor sections were paraffin-embedded and stained for H&E, SOX2, cleaved caspase-3 (CC3), and p27^Kip1^ by the University of Nebraska Medical Center Tissue Sciences Facility. The antibodies used for IHC were: SOX2 (Abcam, ab92494, 1:100), CC3 (Abcam, ab4051, 1:200), and p27^Kip1^ (Cell Signaling, CST83630, 1:700). To quantify the proportion of epithelial cells with protein-of-interest expression, at least 3 distinct, 40X fields were photographed for each independent tumor sample. The ‘count tool’ within Adobe Photoshop (v. 20.0.5) was used to quantitate epithelial cells with positive staining and total cells within the photographed field. Photographs were obtained and cell positivity was scored by an anatomic pathologist unaware of sample designation. Mitotic index (mitoses per 40X field) was used to quantify tumor proliferative rate, and was determined by averaging mitotic figures seen in at least 6, 40X fields for each tumor, using H&E stained slides.

### RNA isolation and cDNA synthesis

RNA was isolated using the RNeasy kit (Qiagen, Germantown, MD). Cells were lysed in provided RLT buffer and spun through Qiashredder columns (Qiagen, Germantown, MD) according to manufacturer’s protocols. RNA was eluted from the column in RNase-free water. cDNA synthesis was performed using the High Fidelity 1st Strand cDNA synthesis kit (Agilent Technologies, La Jolla, CA). Synthesis was performed for 1 h at 42 °C after which the reaction was terminated for 15 min at 70 °C. Primers used in RT-PCR to measure mRNA levels are provided in Table 1. qPCR amplification and quantification was performed on a Bio Rad CFX96 Real time PCR detection system (Bio Rad, Hercules, CA) and detected using RT^2^ SYBR Green qPCR mastermix (Qiagen, Germantown, MD). Transcripts for each gene (control and Dox-treated) were measured in triplicate. Statistical significance was determined using a two-tailed students t-test with a significance threshold of *p* = .05.

**Table 1:** Primers used for qPCR

Name	Forward primer sequence 5′-3’	Reverse primer sequence 5′-3’
GAPDH	ACAGCGACACCCACTCCTCC	GAGGTCCACCACCCTGTTGC
OCT4	GACAACAATGAGAACCTTCAGGAGA	CTGGCGCCGGTTACAGAACCA
NANOG	ATCCAGCTTGTCCCCAAAG	ATTTCATTCGCTGGTTCTGG

## Results

### Elevated levels of SOX2 induce a reversible state of tumor growth arrest

**Fig. 1:**
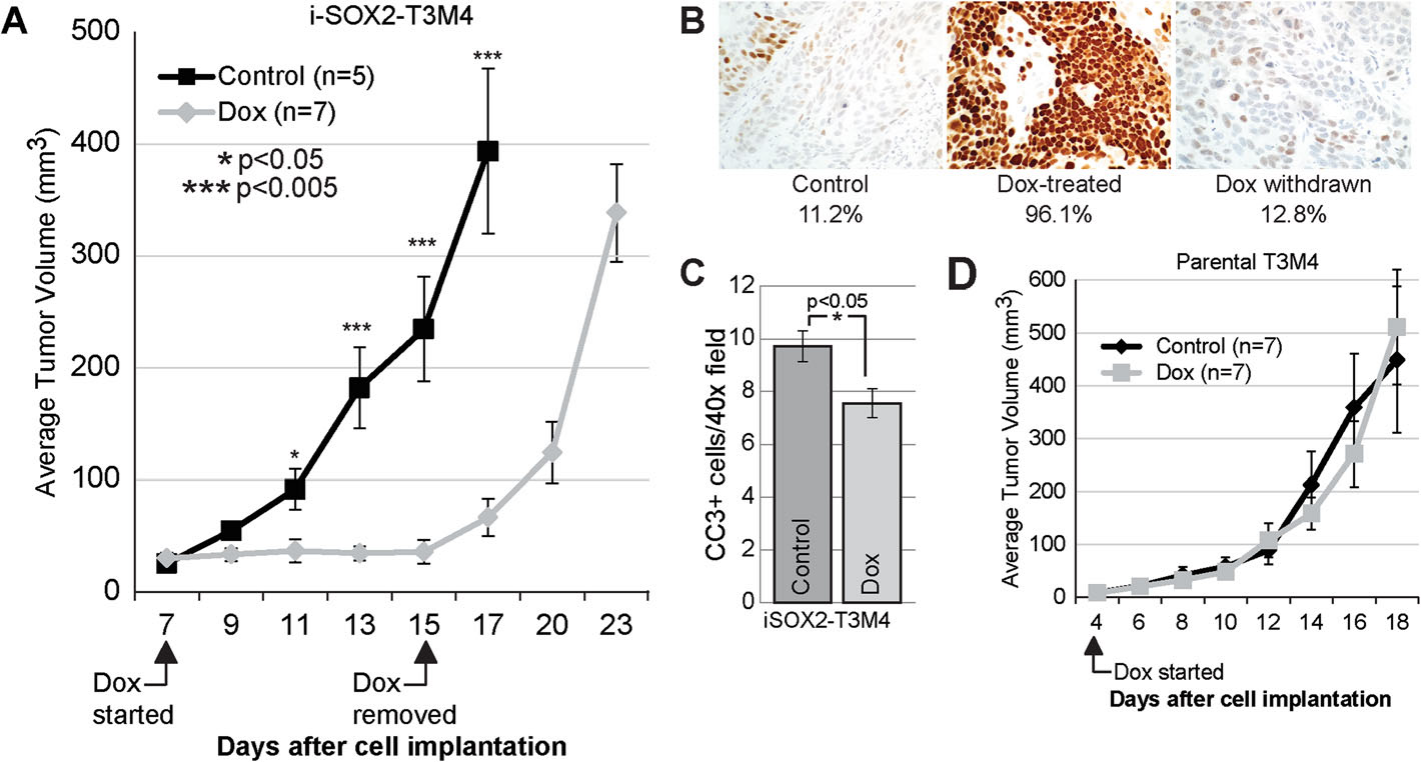
Elevating SOX2 reversibly inhibits i-SOX2-T3M4 tumor growth in vivo. **a** Subcutaneous i-SOX2-T3M4 tumor growth of control and Dox-treated mice. Dox treatment was started and ended at the days indicated. Average tumor volumes are presented for control and Dox-treated groups. **b** IHC analysis of SOX2 expression (percent of cells) in a control tumor, a Dox-growth inhibited tumor, and a Dox-withdrawn tumor. **c** IHC analysis of CC3 in a control and a Dox-treated tumor. **d** Subcutaneous parental T3M4 tumor growth of control and Dox-treated mice. Dox treatment was started and ended at the days indicated. Average tumor volumes are presented for control and Dox-treated groups. Error bars represent standard error of the mean; statistical significance was determined by two-tailed student’s t-test (*p < 0.05, ***p < 0.005)

Previous work in our laboratory demonstrated that elevating SOX2 in vivo with an inducible promoter inhibited the tumor growth of PDAC cells (i-SOX2-T3M4) engineered for elevation of SOX2 [15]. At that time, the fate of the growth-arrested tumors was not determined. Specifically, it was not determined whether the inhibition of tumor growth by elevated SOX2 could be reversed by returning SOX2 to basal levels. To address this question, we engrafted i-SOX2-T3M4 cells subcutaneously into the hind flank of athymic nude mice. Once palpable tumors had formed, mice with size-matched tumors were separated into two groups: control and Dox-treated (to elevate SOX2). As observed previously [15], elevation of SOX2 (Dox-treated mice) halted the growth of i-SOX2-T3M4 tumors; whereas, tumor growth in the control mice continued (Fig. 1a). Importantly, when Dox was removed, tumor growth of the i-SOX2-T3M4 cells resumed rapidly at a rate nearly identical to that of the control tumors. IHC analysis of harvested tumors determined that Dox-treatment elevated SOX2 in vivo, but SOX2 returned to basal levels when Dox was withdrawn from the mice (Fig. 1b). Additionally, we determined that CC3 staining decreased when SOX2 was elevated in vivo (Fig. 1c). Thus, elevating SOX2 does not appear to increase cell death, which is consistent with the rapid resumption of tumor growth when SOX2 returns to endogenous levels. Equally important, in contrast to i-SOX2-T3M4 cells, tumor growth of parental (not engineered) T3M4 cells was unaffected by Dox (Fig. 1d). Together, these results demonstrate that, elevating SOX2 in i-SOX2-T3M4 cells leads to a reversible state tumor growth arrest.

### Elevating SOX2 inhibits the growth of multiple human tumor cell types

**Fig. 2:**
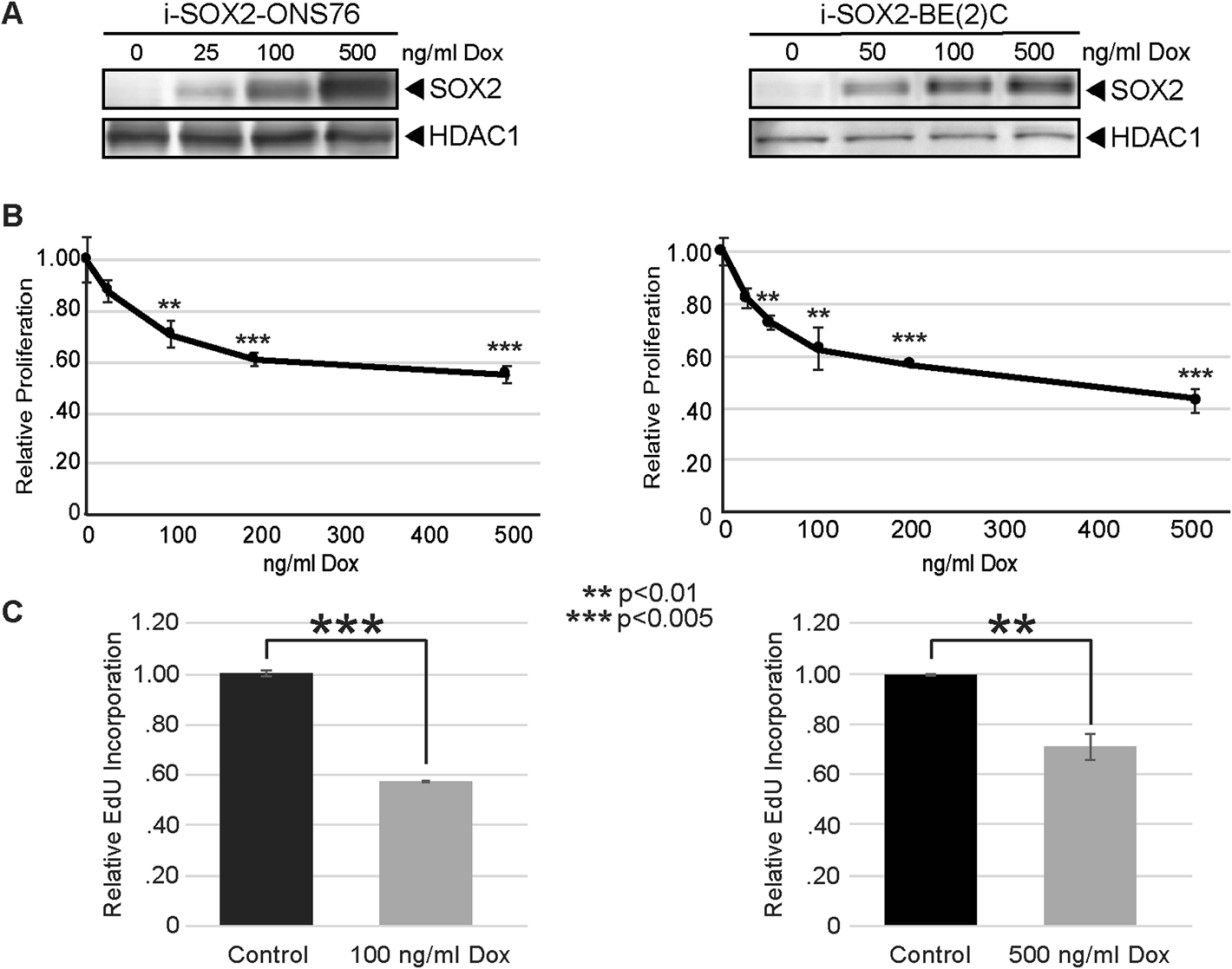
SOX2 elevation inhibits the proliferation of Medulloblastoma and Neuroblastoma cells in vitro. **a** Western blot analysis of whole cell extracts from i-SOX2-ONS76 and i-SOX2-BE(2)C cells cultured for 48 h with the indicated doses of Dox. **b** Proliferation of i-SOX2-ONS76 and i-SOX2-BE(2)C cells was determined by MTT assay following 4 days culture in the presence or absence of Dox at the indicated doses. Error bars represent standard deviation. **c** Relative EdU incorporation of i-SOX2-ONS76 and i-SOX2-BE(2)C cells following 4 days of culture in the presence or absence of Dox at the indicated doses

Our finding that elevating SOX2 in PDAC cells leads to a reversible state of growth arrest led us to test the possibility that this is a general property of SOX2. For this purpose, we engineered four additional tumor cell lines for the inducible elevation of SOX2. Specifically, we engineered two androgen-independent PCa cell lines (DU145 and PC3), one medulloblastoma (MB) cell line (ONS76), and one neuroblastoma (NB) cell line (BE(2)C). Each of these tumor cell lines expresses endogenous SOX2 [21–23]. DU145 is an androgen-independent prostate tumor cell line and PC3 is an androgen-independent PCa cell line with a highly aggressive neuroendocrine phenotype of prostate cancer [24]. ONS76 cells exhibit the properties of sonic hedgehog (subgroup 2) MB [17], and BE(2)C cells are representative of highly aggressive i-type NB cells [23]. Treatment of i-SOX2-ONS76 and i-SOX2-BE(2)C cells with Dox in vitro resulted in a dose-dependent increase in SOX2 (Fig. 2a). Similarly, treatment with Dox increased the expression of SOX2 in i-SOX2-DU145 and i-SOX2-PC3 cells (Figure S1). Moreover, in agreement with our previous findings with other tumor cells [14–16], elevating SOX2 inhibited the in vitro proliferation of i-SOX2-DU145, i-SOX2-PC3 PCa, i-SOX2-ONS76, and i-SOX2-BE(2)C cells (Fig. 2b, Figure S1B). The reduction in cell proliferation as measured by MTT assay was confirmed by performing an Edu incorporation assay (Fig. 2c, Figure S1C). Importantly, treatment of the four corresponding unengineered, parental tumor cell lines with Dox did not significantly affect their proliferation in vitro (Figure S2). Altogether, these findings demonstrate that elevating SOX2 in multiple tumor cell lines representing three additional tumor types (androgen-independent PCa, MB and NB) inhibits cell proliferation in vitro.

To determine whether elevation of SOX2 induces a reversible state of tumor growth arrest in additional tumor types, we tested two PCa lines and one MB line. Using the protocol used earlier for i-SOX2-T3M4 cells, i-SOX2-DU145 PCa cells were engrafted subcutaneously into the hind flank of athymic nude mice. When palpable tumors were present on day 6, mice with size-matched tumors were separated into control and Dox-treated groups.

**Fig. 3:**
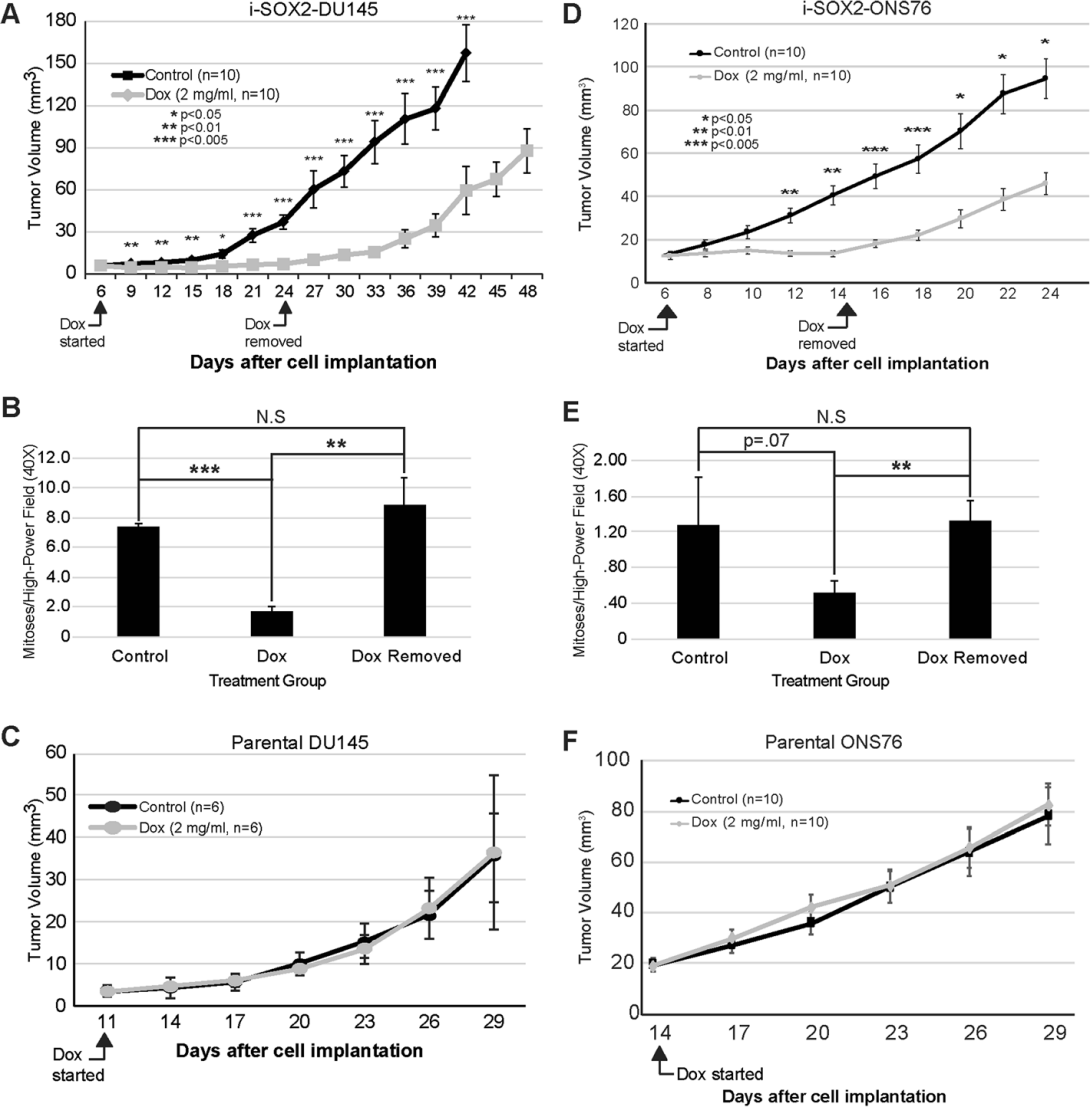
Elevating SOX2 reversibly inhibits the growth of i-SOX2-DU145 and i-SOX2-ONS76 xenografts. **a** Subcutaneous i-SOX2-DU145 tumor growth of control and Dox-treated mice. Dox treatment was started and ended at the days indicated. Average tumor volumes are presented for control and Dox-treated groups. **b** Subcutaneous i-SOX2-ONS76 tumor growth of control and Dox-treated mice. Dox treatment was started and ended at the days indicated. Average tumor volumes are presented for control and Dox-treated groups. **c** Mitotic figures per high-power field in i-SOX2-DU145 tumors. **d** Mitotic figures per high-power field in i-SOX2-ONS76 tumors. **e** Subcutaneous parental DU145 tumor growth of control and Dox-treated mice. Dox treatment was started and stopped at the days indicated. Average tumor volumes are presented for control and Dox-treated groups. **f** Subcutaneous parental ONS76 tumor growth of control and Dox-treated mice. Dox treatment was started and stopped at the days indicated. Average tumor volumes are presented for control and Dox-treated groups. Error bars represent standard error of the mean; statistical significance was determined by two-tailed student’s t-test (*p < 0.05, **p < 0.01, ***p < 0.005)

Administration of Dox robustly inhibited the growth of i-SOX2-DU145 tumors compared to the control tumors until Dox was removed on day 24 (Fig. 3a). Similar to i-SOX2-T3M4 tumors, tumor growth of i-SOX2-DU145 cells resumed rapidly when Dox was removed. In addition, IHC staining demonstrated that treatment with Dox led to a dramatic increase in the expression of SOX2, which returned to basal levels after Dox was removed (Figure S3A). As expected, treatment with Dox led to a large decrease in the number of mitotic indices, which returned to levels observed in control tumors when Dox was removed (Fig. 3b). Again, administration of Dox to parental, unengineered DU145 xenografts did not alter tumor growth (Fig. 3c).

Next, we examined whether elevating SOX2 in vivo inhibited the growth of i-SOX2-PC3 xenografts. Once palpable tumors had formed (day 17) one group of mice was treated with Dox resulting in inhibition of tumor growth (Figure S4A). Again, when Dox was removed (day 32) tumor growth of i-SOX2-PC3 cells resumed at a rate virtually identical to that of the control mice. Additionally, treatment of parental, unengineered PC3 cells did not affect tumor growth (Figure S4B). Finally, we examined how elevating SOX2 in the MB cell line ONS76 (i-SOX2-ONS76) affected tumor growth. As observed for the other SOX2 engineered tumor cell lines, subcutaneous tumor growth of i-SOX2-ONS76 cells was strongly inhibited when tumor-bearing mice were treated with Dox (day 6), and it resumed rapidly when Dox was removed (day 14) (Fig. 3d). Similar to tumors formed by i-SOX2-T3M4 and i-SOX2-DU145 cells, IHC analysis demonstrated that treatment of i-SOX2-ONS76 tumors with Dox led to a substantial increase in SOX2 staining, which returned to basal levels when Dox was removed (Figure S3B). Additionally, Dox treatment reduced the number of mitotic indices observed, which approached, but did not reach, statistical significance (Fig. 3e). However, when Dox was removed the number of mitotic indices reached levels that were not statistically different from those observed in the control group (Fig. 3e). Importantly, Dox treatment did not affect the tumor growth of unengineered, parental ONS76 cells (Fig. 3f). Collectively, our findings demonstrate that elevating SOX2 in vivo leads to a reversible state of tumor growth arrest in four different tumor cell lines representing three different tumor types.

### Elevation of SOX2 downregulates a broad spectrum of the cell cycle machinery while upregulating p27^Kip1^

**Fig. 4:**
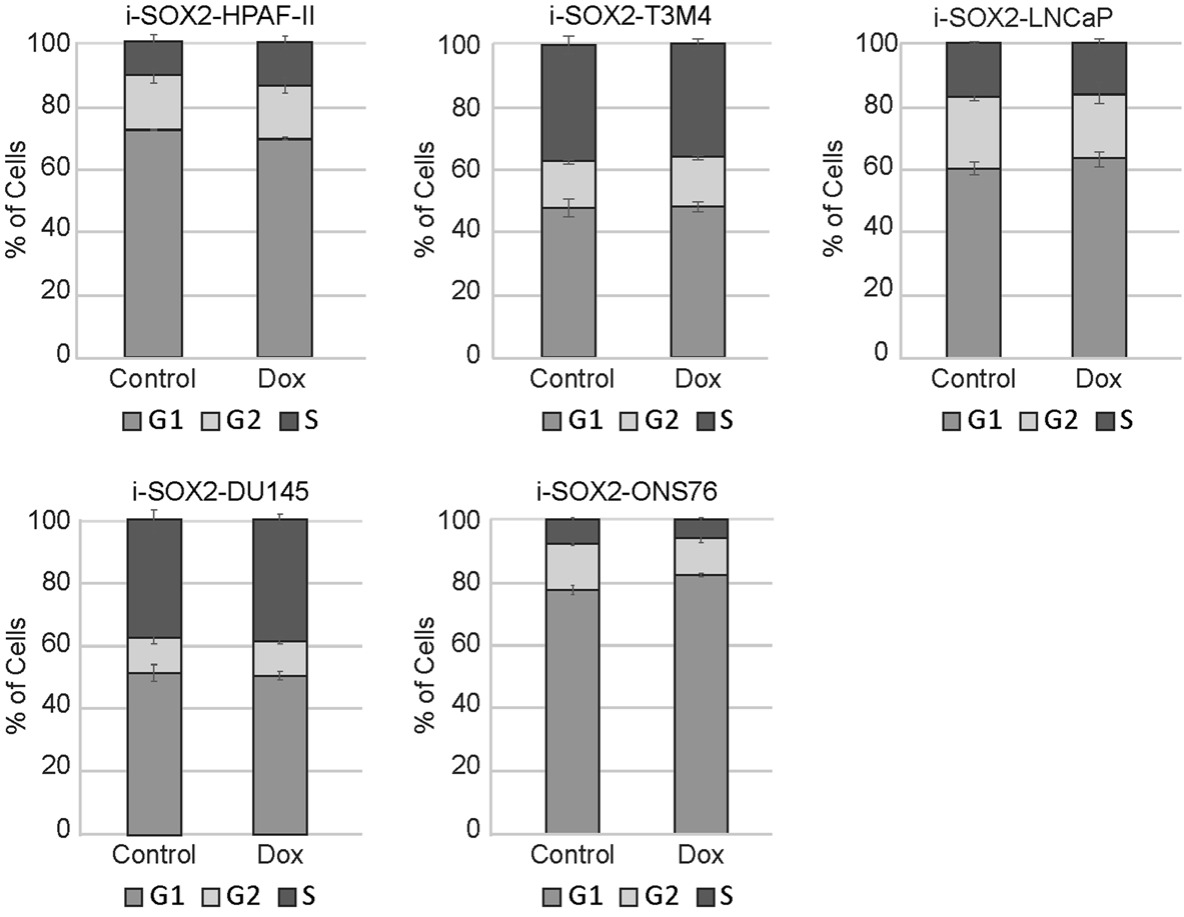
Elevating SOX2 does not alter cell cycle distribution of tumor cells despite growth inhibition. Cell cycle analysis for each of the five cell lines was performed by flow cytometry after 4 days culture in the presence or absence of Dox at the doses indicated. Error bars represent standard deviation

The studies described above indicate that elevating SOX2 leads to growth inhibition both in vitro and in vivo in many different tumor cell lines representing a diverse group of human tumor types. However, the mechanisms by which SOX2 inhibits growth when elevated from an inducible promoter have not been determined. To begin to address this deficiency, we examined whether elevation of SOX2 in five different tumor cell lines alters the fraction of cells in the G1, S, and G2/M phases of the cell cycle. For this purpose, the cells were cultured in the presence or absence of Dox for 4 days, the same duration used to determine in vitro growth inhibition. For these studies, we focused on five i-SOX2 tumor cell lines: i-SOX2-LNCaP, i-SOX2-DU145, i-SOX2-T3M4, i-SOX2-HPAF-II, and i-SOX2-ONS76. i-SOX2-T3M4, i-SOX2-HPAF-II, and i-SOX2-LNCaP have been described previously, and i-SOX2-DU145, and i-SOX2-ONS76 are described above [15, 16]. Although elevation of SOX2 inhibits the in vitro growth of all five tumor cell lines, analysis of the cell cycle distribution of the cells by the Telford method indicated that elevating SOX2 had little or no effect on their cell cycle distribution (Fig. 4). Importantly, these results strongly contrast with the effects of a CDK4/6 inhibitor (palbociclib), which causes G1 arrest (Figure S5). Additionally, although we did not observe a change in the sub-G1 populations of the cells treated with Dox (data not shown), it is possible that elevated SOX2 has some effect on cell survival. Furthermore, for one of these tumor cell lines, i-SOX2-ONS76, we examined whether elevating SOX2 altered the cell cycle distribution at earlier time points. Similar to the results after 4 days of treatment with Dox, there was little or no change in the cell cycle distribution of the cells 1 day after treatment with Dox (Figure S6). However, we did observe a small decrease in the proportion of the cells in the G2 phase after treatment with Dox for 2 days.

The finding that elevating SOX2 did not appear to dramatically alter the cell cycle distribution of the cells led us to use a second method to probe for changes in their cell cycle distribution. For this purpose, we used the fluorescence ubiquitination cell cycle indicator (FUCCI) method, which relies on the expression of two cell cycle regulated proteins: cdt1 (only present in the G1 phase of the cell cycle) and geminin (only present in the S, G2 and M phases of the cell cycle) [20]. Similar to the results obtained by the Telford method, elevating SOX2 in i-SOX2-DU145, i-SOX2-T3M4, i-SOX2-HPAF-II, and i-SOX2-LNCaP cells had little or no effect on cell cycle distribution (Figure S7). However, elevating SOX2 in i-SOX2-ONS76 cells led to a moderate increase in the S/G2/M population with a corresponding decrease in the G1 population. Currently, it is unclear why the Telford method and the FUCCI method yielded different readouts for the effect of elevating SOX2 in i-SOX2-ONS76 cells. Overall, our findings indicate that elevating SOX2 in four of the five tumor cell lines had little or no effect on the cell cycle distribution of the cells even though elevating SOX2 decreases their proliferation.

**Fig. 5:**
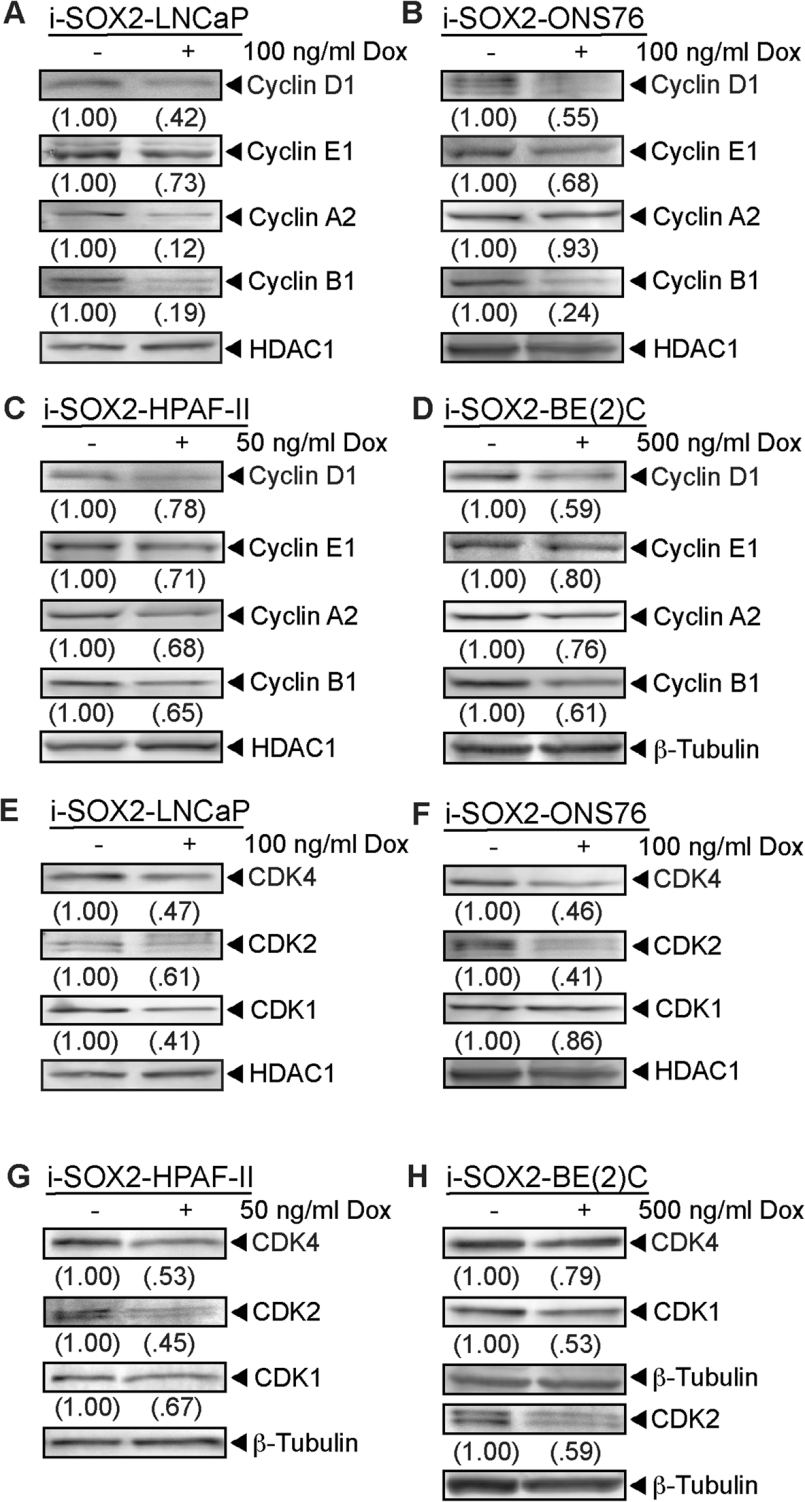
SOX2 elevation decreases the expression of multiple cyclins and CDKs. **a-d** Western blot analyses of the cyclins indicated in whole cell extracts harvested from i-SOX2-LNCaP, i-SOX2-ONS76, i-SOX2-HPAF-II, and i-SOX2-BE(2)C cells after 48 h culture in the presence or absence of Dox at the concentration indicated. **e-h** Western blot analyses of the CDKs indicated in whole cell extracts harvested from i-SOX2-LNCaP, i-SOX2-ONS76, i-SOX2-HPAF-II, and i-SOX2-BE(2)C cells after 48 h culture in the presence or absence of Dox at the concentration indicated. Western blot analyses of cyclins and CDKs were repeated using separate whole extracts for each cell line and similar results were obtained

Our finding that the cell cycle distribution of four tumor cell lines exhibited little or no change when SOX2 was elevated raised the possibility that elevating SOX2 acts by altering the cell cycle machinery for all phases of the cell cycle. To test this possibility, we initially examined the expression of multiple cyclins in control and Dox-treated i-SOX2-LNCaP cells. Intriguingly, we determined that elevating SOX2 in i-SOX2-LNCaP cells led to significant decreases in the expression of cyclin D1, cyclin A2, and cyclin B1 with a moderate decrease in cyclin E1 levels (Fig. 5a). Moreover, elevating SOX2 in i-SOX2-DU145 cells decreased the expression of cyclin D1, cyclin E1, cyclin A2, and cyclin B1 (Figure S8). Significantly, similar results were observed in three additional SOX2 engineered cell lines. Similar to the results with i-SOX2-LNCaP and i-SOX2-DU145 cells, SOX2 elevation in i-SOX2-ONS76 MB decreased the levels of cyclin D1, cyclin E1, and cyclin B1, whereas cyclin A2 was not altered significantly (Fig. 5b). SOX2 elevation in i-SOX2-HPAF-II and i-SOX2-BE(2)C cells also reduced the expression of cyclin D1, cyclin E1, cyclin A2, and cyclin B1 (Fig. 5c, d). Finally, we determined that elevating SOX2 in i-SOX2-T3M4 cells decreased the expression of cyclin D1, A2, and B1, but did not decrease the expression of cyclin E1 (Figure S9).

**Fig. 6:**
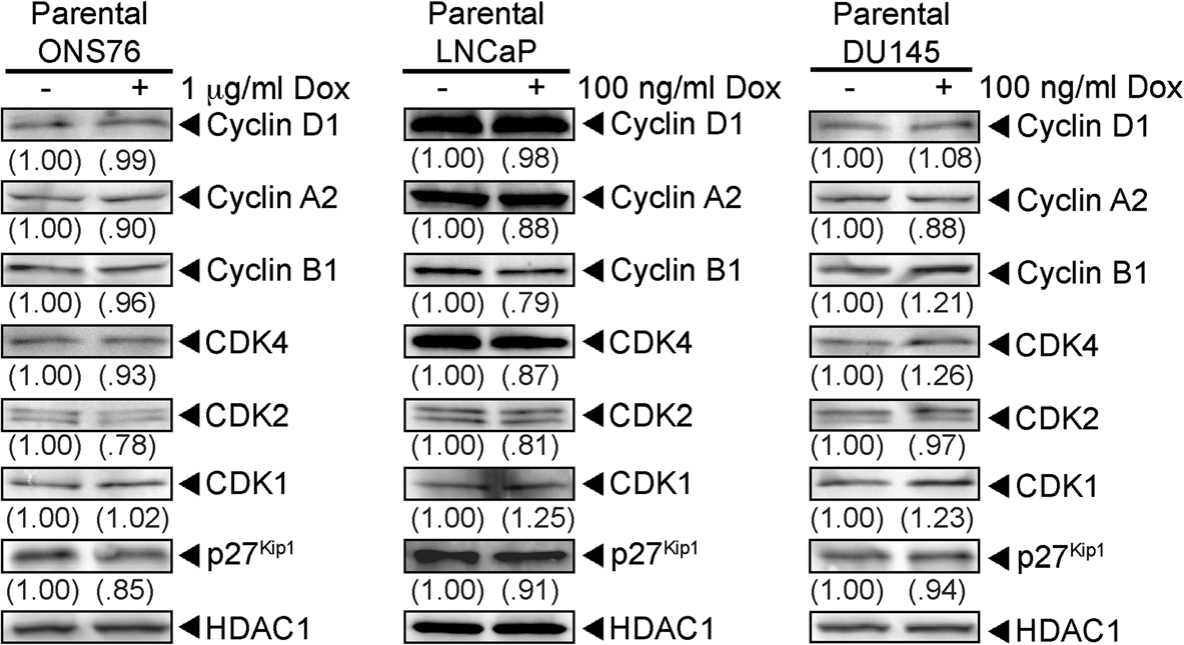
Dox treatment does not alter cell cycle machinery in parental ONS76, LNCaP, and DU145 cells. Western blot analyses of cyclins and CDKs in whole cell extracts from ONS76, LNCaP, and DU145 whole cell extracts that were harvested after 48 h culture in the presence or absence of Dox at the concentrations indicated

To extend our studies of the cell cycle machinery, we examined how SOX2 elevation affected the expression of cyclin-dependent kinases (CDKs). Elevating SOX2 in i-SOX2-LNCaP, i-SOX2-BE(2)C, and i-SOX2-DU145 cells decreased the expression of CDK4, CDK2, and CDK1 (Fig. 5e, h, Figure S8), whereas elevating SOX2 in i-SOX2-ONS76 MB cells and i-SOX2-HPAF-II cells decreased the expression of CDK4 and CDK2, but had little or no effect on CDK1 (Fig. 5f and g). In the case of i-SOX2-T3M4 cells only CDK4 was reduced significantly (Figure S9). As a control, we determined that Dox did not alter the expression of cyclins or CDKs in three of the parental, unengineered tumor cell lines: ONS76, LNCaP, and DU145 (Fig. 6). Collectively, these studies in multiple tumor cell lines, representing four human tumor types, demonstrate that elevating SOX2 decreases expression of cyclins and/or CDKs associated with each phase of the cell cycle. Significantly, we observed moderate decreases in multiple cyclins and CDKs in many tumor cell types, rather than observing large decreases in one or a few cyclins and/or CDKs. Consistent with these findings, we did not observe large differences in the cell cycle distribution in at least four of the five cell lines when SOX2 was elevated (Fig. 4, Figure S7) despite clear growth inhibition.

**Fig. 7:**
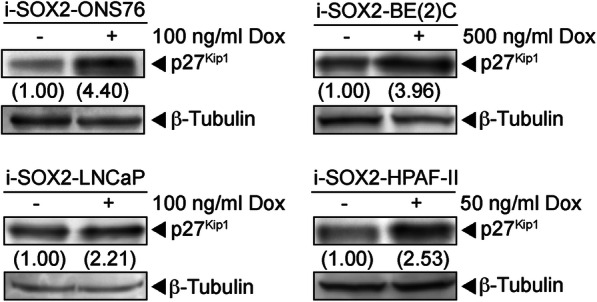
SOX2 elevation modulates the expression of p27^Kip1^. Western blot analyses of p27^Kip1^ in i-SOX2-LNCaP, i-SOX2-ONS76, i-SOX2-HPAF-II, and i-SOX2-BE(2)C whole cell extracts that were harvested after 48 h culture in the presence or absence of Dox at the indicated concentrations. Western blot analyses of p27^Kip1^ were repeated using separate whole extracts for each cell line and similar results were obtained

In addition to examining changes in cyclins and CDKs we examined how elevating SOX2 affected the expression of p27^Kip1^ levels, because p27^Kip1^ can inhibit multiple cyclin-CDK complexes [25]. Western blot analysis of whole cell extracts from i-SOX2-LNCaP, i-SOX2-ONS76, i-SOX2-BE(2)C, and i-SOX2-HPAF-II cells demonstrated that, in each case, elevating SOX2 increased the expression of p27^Kip1^ (Fig. 7). Intriguingly, elevating SOX2 in i-SOX2-DU145 cells in vitro did not alter the total levels of p27^Kip1^ (data not shown). However, when SOX2 was elevated in vivo in i-SOX2-DU145 cells, there was a substantial increase in nuclear p27^Kip1^ staining, which was reversed when Dox was removed (Figure S10). Moreover, although less pronounced, there was an increase in nuclear p27^Kip1^ staining when i-SOX2-ONS76 tumors were treated with Dox, which was reversed when Dox was removed (Figure S10). Given the known function of p27^Kip1^, this finding is consistent with growth inhibition when SOX2 is elevated and the need for p27^Kip1^ to exit the nucleus during cell cycle progression [26, 27]. Taken together, our studies demonstrate that elevating SOX2 in tumor cell lines representing four human tumor types leads to downregulation of a broad spectrum of the cell machinery required for progression through the G1, S, and G2/M phases of the cell cycle, while upregulating or altering the subcellular localization of p27^Kip1^.

Finally, we examined the effects of elevating SOX2 on the expression of two other stem cell transcription factors: OCT4 and NANOG. In i-SOX2-ONS76 cells, *NANOG* expression was below the level of detection by qPCR (40 cycles), and there was no statistically significant change in the expression of *OCT4* (Figure S11). In contrast, in i-SOX2-LNCaP cells there was no statistically change in the expression of *OCT4*, but *NANOG* expression decreased approximately 60% (Figure S11). These findings differ from our earlier work where we determined that elevating Sox2 in mouse F9 embryonal carcinoma cells led to a reduction in the expression of both *Oct4* and *Nanog* mRNA [28]. Consequently, it appears that the transcriptional circuitries that control the expression of *OCT4* and *NANOG* differ in different cell types.

## Discussion

The studies described in this report provide new insights into the function of SOX2 in a diverse set of human tumor cells. We show that SOX2 elevation with the aid of an inducible promoter inhibits the proliferation of four tumor cell lines. These findings, together with our earlier work [14–16], indicate that elevating SOX2 in a diverse set of tumor cell lines representing six types of human cancer, leads to growth inhibition. Similar to our studies, elevation of SOX2 has also been reported to inhibit the growth of gastric tumor cells [29]. In contrast to the tumor types used in our studies, where SOX2 expression correlates with shorter patient survival [10, 23, 30] SOX2 expression in gastric cancer correlates with longer patient survival [29, 31]. Thus, elevating SOX2 in tumor cells appears to cause growth inhibition independently of the prognostic significance of SOX2. In addition, we demonstrate in four tumor cell lines, representing three different tumor types, that elevating SOX2 leads to a reversible state of tumor growth arrest. In our system, tumor growth is arrested when SOX2 is elevated, yet tumor growth resumes rapidly when Dox is removed and SOX2 returns to endogenous levels. Finally, we demonstrate in six different cell lines, representing four different tumor types, that SOX2 elevation decreases multiple proteins that regulate progression through each phase of the cell cycle. Thus, SOX2 appears to function as a master regulator that controls a broad spectrum of the cell cycle machinery.

The finding that elevating SOX2 inhibits the proliferation of tumor cells parallels the effects of elevated levels of SOX2 in normal cells. Earlier studies demonstrated that elevated levels of SOX2 inhibit cell proliferation in many developing tissues, including in the developing central nervous system, spinal cord, stomach, esophagus, and lung [8, 9]. This was initially shown in the developing central nervous system [32] and shortly thereafter in the postnatal eye [33]. The relationship between elevated levels of SOX2 and quiescence has also been described in the slowly cycling radial glia cells in embryonic cortex and cells in the subventricular zone of the brain [8]. Importantly, a causal relationship between high levels of SOX2 and the decreased proliferation of cells in developing tissues was established by knocking down and overexpressing SOX2 in SOX2^high^ and SOX2^low^ cells, respectively [9]. Subsequently, overexpression studies and expression of a dominant negative form of SOX2 further demonstrated a cause-and-effect relationship between SOX2 levels and the proliferative status of SOX2^low^ and SOX2^high^ cells in the developing stomach and spinal cord [9]. Additionally, quiescent inner pillar cells in the auditory epithelium have been shown to express high levels of SOX2 and p27^kip1^ [34]. Significantly, knocking out SOX2 in these cells increased proliferation and decreased the expression of p27^kip1^.

In addition to the work from our laboratory, including the studies reported here, there are a growing number of reports that strongly support the conclusion that tumor cells hijack the normal growth inhibitory effects of elevated SOX2 for their own needs. In the case of human lung tumor cells, intracardiac injection led to dissemination to lung where the cells could remain dormant for months [13]. Significantly, these quiescent cells were found to express higher levels of SOX2 than the starting parental cell population. Moreover, when isolated, these long-term quiescent cells exhibited a higher frequency of tumor-initiating cells than the original parental population. Similarly, in a mouse model of SHH group medulloblastoma, SOX2 expression within the tumor was found to be restricted to a small quiescent subpopulation [11]. Importantly, these quiescent SOX2^high^ cells were found to resist chemotherapies and were the first cells to repopulate the tumor following withdrawal of drug treatment. Additionally, colorectal tumor cells that endogenously express higher levels of SOX2 proliferate more slowly, exhibit higher chemoresistance, and possess a higher tumor-initiating capacity than the SOX2^low^ tumor cell population [12]. Altogether, it is evident that elevating SOX2 above levels required for proliferation leads to reduction in proliferation of both normal cells and tumor cells. We believe this is a fundamental property of SOX2.

Given the need to carefully control the levels of SOX2 in both normal and tumor cells, it is not surprising that SOX2 is regulated by a highly diverse set of regulatory mechanisms that control its transcription, translation, subcellular location, transcriptional activity, and stability. This includes the use of over a dozen distal enhancers, at least 16 miRNAs, six lncRNAs, and six different post-translational modifications distributed over 13 amino acid residues [10] Furthermore, we have shown that SOX2 is part of a highly integrated transcriptional network where SOX2 interacts with many other master regulators [10, 35–37], which helps explain why changes in the levels of SOX2 have such consequential effects on cell function [37].

Although our studies and the work of others indicate that elevating SOX2 can lead to growth inhibition, many studies, including our own, have shown that SOX2 is required for the proliferation of tumor cells. Knockdown studies have demonstrated that SOX2 is required for growth of cells from over 20 different human cancers [10]. Interestingly, our earlier studies with PDAC cells demonstrated that both elevating SOX2 and knocking down SOX2 inhibit tumor growth [15]. Thus, we proposed that SOX2 levels in tumor cells are optimized to maximize tumor growth – too little or too much SOX2 reduces tumor growth [5, 10]. Importantly, our studies indicate that absolute levels of SOX2 are not the sole determinant for how cells respond to SOX2. We have determined that elevating SOX2 inhibits the proliferation of tumor cells that express widely different levels of endogenous SOX2. For example, DU145 cells express substantially higher levels of endogenous SOX2 than LNCaP cells [22], yet the proliferation of both tumor cell lines is inhibited when SOX2 is overexpressed from an inducible promoter. Additionally, there is ample evidence that SOX2 levels rise during tumor progression. However, this is likely to occur in the context of other genetic and epigenetic changes. For example, knockdown of RB1 and p53 leads to significant elevation of SOX2 in prostate tumor cells without causing growth inhibition in vitro or in vivo [38, 39]. Consequently, changes in other genes, which can counterbalance the growth inhibitory effects of elevated SOX2, enable SOX2 levels to rise during tumor progression. Thus, the SOX2 “Goldilocks” zone for tumor cell proliferation appears to be highly context dependent.

Interestingly, and in contrast to our findings, several earlier studies reported that stable overexpression of SOX2 increased the proliferation of tumor cells [10]. This led to the belief that elevation of SOX2 increases tumor cell growth both in vitro and in vivo. However, our studies clearly show that increasing SOX2 on its own in over 13 tumor cell lines without changes in other genes, such as RB1 and p53, inhibits the proliferation of tumor cells. Significantly, we have observed growth inhibition in every tumor cell line tested in this laboratory when SOX2 is elevated, including 7 additional tumor cell lines (data not shown) that we have not yet reported. As discussed elsewhere [5, 10], the different results observed with stable overexpression and inducible overexpression of SOX2 is due to a fundamental difference in experimental design. In our studies, the effects of elevated SOX2 are examined only after the cells have been fully engineered. In contrast, during the engineering of tumor cells for stable overexpression, SOX2 is elevated during the drug selection step. Thus, cells that are growth inhibited due to elevated SOX2 are lost early during drug selection.

Significantly, our current work also describes a new model in which tumor growth can be halted and restarted by first increasing then decreasing the levels of SOX2. Specifically, we demonstrate for the first time that elevating SOX2 in vivo leads to a reversible state of tumor growth arrest. In addition to providing a novel model system to characterize mechanisms of tumor cell quiescence, our reversible in vivo model of tumor growth arrest could be used to screen drugs for their ability to effectively target and eradicate quiescent tumor cells in vivo. This could help address a major unmet need in the treatment of cancer. It is widely recognized that current chemotherapies target proliferating cells, but spare quiescent and long-term dormant tumor-initiating cells, which are able to drive tumor recurrence.

Finally, our studies provide new and surprising insights into the molecular mechanisms by which SOX2 inhibits the proliferation of tumor cells. We show for the first time that elevating SOX2 in tumor cells representing four different tumor types decreases the expression of a broad spectrum of cell cycle machinery, in particular cyclins and CDKs that control the transition of each phase of the cell cycle. Our finding that this occurs in many different tumor cell lines leads us to propose that elevating SOX2 inhibits the growth of tumor cells by activating a well-conserved mechanism, rather than using a different mechanism in each tumor type. Our studies also show that elevating SOX2 increases the expression of p27^Kip1^ in four out of the five tumor cell lines studied. Increased expression of p27^Kip1^ upon elevation of SOX2 is potentially significant for several reasons. First, as described earlier, SOX2 has been shown to elevate p27^Kip1^ expression during normal tissue homeostasis [34]. Second, p27^Kip1^ is able to inhibit multiple cyclin-CDK complexes [25]. Additionally, high expression of p27^Kip1^ has been implicated in the maintenance of dormant tumor cells [40, 41]. Thus, in the future it will be important to determine the molecular events triggered by elevated SOX2 that cause downregulation of the cell cycle machinery while increasing the expression of p27^Kip1^. Understanding the molecular mechanisms involved may help identify new strategies for targeting quiescent tumor cells.

## Conclusion

The studies described in this report, in addition to our earlier work [14–16], demonstrate that elevating SOX2 in vitro inhibits the proliferation of a large number of human tumor cell types. Additionally, elevating SOX2 in vivo in four tumor cell lines, representing three human tumor types, leads to a reversible state of tumor growth arrest. Lastly, we show that elevating SOX2 leads to the downregulation of a broad spectrum of cyclins and cyclin-dependent kinases (CDKs), which inhibits progression through all phases of the cell cycle. Collectively, our SOX2-inducible tumor studies provide a novel model system for investigating the molecular mechanisms by which elevated levels of SOX2 restrict cell proliferation.

## Supplementary information

**Additional file 1: Figure S1.** Elevating SOX2 inhibits the in vitro proliferation of i-SOX2-DU145 and i-SOX2-PC3 cells. **A.** Western blot analysis of SOX2 in whole cell extracts from i-SOX2-DU145 and i-SOX2-PC3 cells cultured for 48 h with Dox at the indicated doses. **B.** Cell proliferation of i-SOX2-DU145 and i-SOX2-PC3 cells were determined by MTT assay following 4 days culture in the presence or absence of Dox at the indicated doses. Error bars represent standard deviation. **C.** Relative EdU incorporation of i-SOX2-DU!45 and i-SOX2-PC3 cells following 4 days of culture in the presence or absence of Dox at the doses indicated.

**Additional file 2: Figure S2.** Dox treatment does not affect the proliferation of parental tumor cell lines. Proliferation of DU145, PC3, ONS76, and BE(2)C cells were determined by MTT assay following 4 days culture in the presence or absence of Dox at the indicated doses. Error bars represent standard deviation.

**Additional file 3: Figure S3.** Dox treatment increases SOX2 expression in i-SOX2-DU145 and i-SOX2-ONS76 tumors in vivo. Immunohistochemical analysis of SOX2 expression in **(A)** i-SOX2-DU145 tumors and **(B)** i-SOX2-ONS76 tumors.

**Additional file 4: Figure S4.** Elevating SOX2 in vivo reversibly inhibits the growth of i-SOX2-PC3 tumors. **A.** Subcutaneous i-SOX2-PC3 tumor growth of control and Dox-treated mice. Dox treatment was started and ended at the days indicated. Average tumor volumes are presented for control and Dox-treated groups. **B.** Subcutaneous parental PC3 tumor growth of control and Dox-treated mice. Dox treatment was started and stopped at the days indicated. Average tumor volumes are presented for control and Dox-treated groups. Error bars represent standard error of the mean; statistical significance was determined by two-tailed student’s t-test (*p<0.05).

**Additional file 5: Figure S5.** Palbociclib induces G1 cell cycle arrest. Cell cycle analysis was performed by flow cytometry after 4 days treatment with DMSO (control) and 1 μM Palbociclib. Error bars represent standard deviation.

**Additional file 6: Figure S6.** Elevating SOX2 does not dramatically alter the cell cycle distribution of i-SOX2-ONS76 cells at the one and two day time point. I-SOX2-ONS76 cells were subjected to cell cycle analysis after 24 or 48 h culture in the presence or absence of 100 ng/ml Dox. Error bars represent standard deviation.

**Additional file 7: Figure S7.** Elevating SOX2 does not significantly alter the cell cycle distribution of the majority of tumor cell lines examined. Cell cycle analysis was performed by flow cytometry using the FastFUCCI system after 4 days in the presence or absence of Dox at doses the indicated.

**Additional file 8: Figure S8.** Elevating SOX2 in i-SOX2-DU145 cells decreases the expression of multiple cyclins and CDKs. Western blot analysis of i-SOX2-DU145 whole cell extracts harvested after 48 h growth in the presence or absence of 100 ng/ml Dox.

**Additional file 9: Figure S9.** Elevating SOX2 in i-SOX2-T3M4 cells decreases the expression of cyclins and CDKs. Western blot analysis of i-SOX2-T3M4 whole cell extracts harvested after 48 h growth in the presence or absence of 300 ng/ml Dox.

**Additional file 10: Figure S10.** Elevating SOX2 in i-SOX2-DU145 and i-SOX2-ONS76 cells increases p27^Kip1^ nuclear localization. Immunohistochemical analysis of p27^Kip1^ expression in control, Dox-treated, and Dox-removed i-SOX2-DU145 and i-SOX2-ONS76 tumors.

**Additional file 11: Figure S11.** Elevating SOX2 does not alter the expression of *OCT4* in i-SOX2-ONS76 or i-SOX2-LNCaP cells, but decreases *NANOG* expression in i-SOX2-LNCaP cells. RT-qPCR analysis of *OCT4* and *NANOG* expression in mRNA from i-SOX2-ONS76 and i-SOX2-LNCaP cells were cultured in the presence or absence of 100 ng/ml Dox for 48 h.

**Additional file 12: Figure 2A western blots.** The original, full-length membrane images of western blot data in Figure 2A.

**Additional file 13: Figure 5A-D western blots.** The original, full-length membrane images of western blot data in Figure 5A-D. Additional bands are due to repeated stripping and reprobing of the membrane.

**Additional file 14: Figure 5E-H western blots.** The original, full-length membrane images of western blot data in Figure 5E-H. Additional bands are due to repeated stripping and reprobing of the membrane.

**Additional file 15: Figure 6 western blots.** The original, full-length membrane images of western blot data in Figure 6. Additional bands are due to repeated stripping and reprobing of the membrane.

**Additional file 16: Figure 7 western blots.** The original, full-length membrane images of western blot data in Figure 7. Additional bands are due to repeated stripping and reprobing of the membrane.

**Additional file 17: Figure S1A western blots.** The original, full-length membrane images of western blot data in Figure S1A.

**Additional file 18: Figure S8 western blots.** The original, full-length membrane images of western blot data in Figure S8. Additional bands are due to repeated stripping and reprobing of the membrane.

**Additional file 19: Figure S9 western blots.** The original, full-length membrane images of western blot data in Figure S9. Additional bands are due to repeated stripping and reprobing of the membrane.

## Data Availability

The cell lines used in this study and the data that support the findings of this study are available from the corresponding author upon reasonable requests.

## Abbreviations

CC3: Cleaved Caspase 3
CDK: Cyclin-Dependent Kinase
Dox: Doxycycline
H&E: Hematoxylin and Eosin
IHC: Immunohistochemistry
MB: Medulloblastoma
MTT: 3-(4,5-Dimethylthiazol-2-yl)-2,5-diphenyltetrazolium bromide
NB: Neuroblastoma
PBS: Phosphate Buffered Saline
PCa: Prostate Cancer
PDAC: Pancreatic Ductal Adenocarcinoma
RB1: Retinoblastoma
RIPA: Radioimmunoprecipitation Assay buffer
RT-qPCR: Reverse Transcription Quantitative Polymerase Chain Reaction
SHH: Sonic Hedgehog
