# Supplementary figures and images for: Tumor quiescence: elevating SOX2 in diverse tumor cell types downregulates a broad spectrum of the cell cycle machinery and inhibits tumor growth
Source: BMC Cancer. 2020 Oct 1;20:941. doi: 10.1186/s12885-020-07370-7 (PMC7528478; doi:10.1186/s12885-020-07370-7)

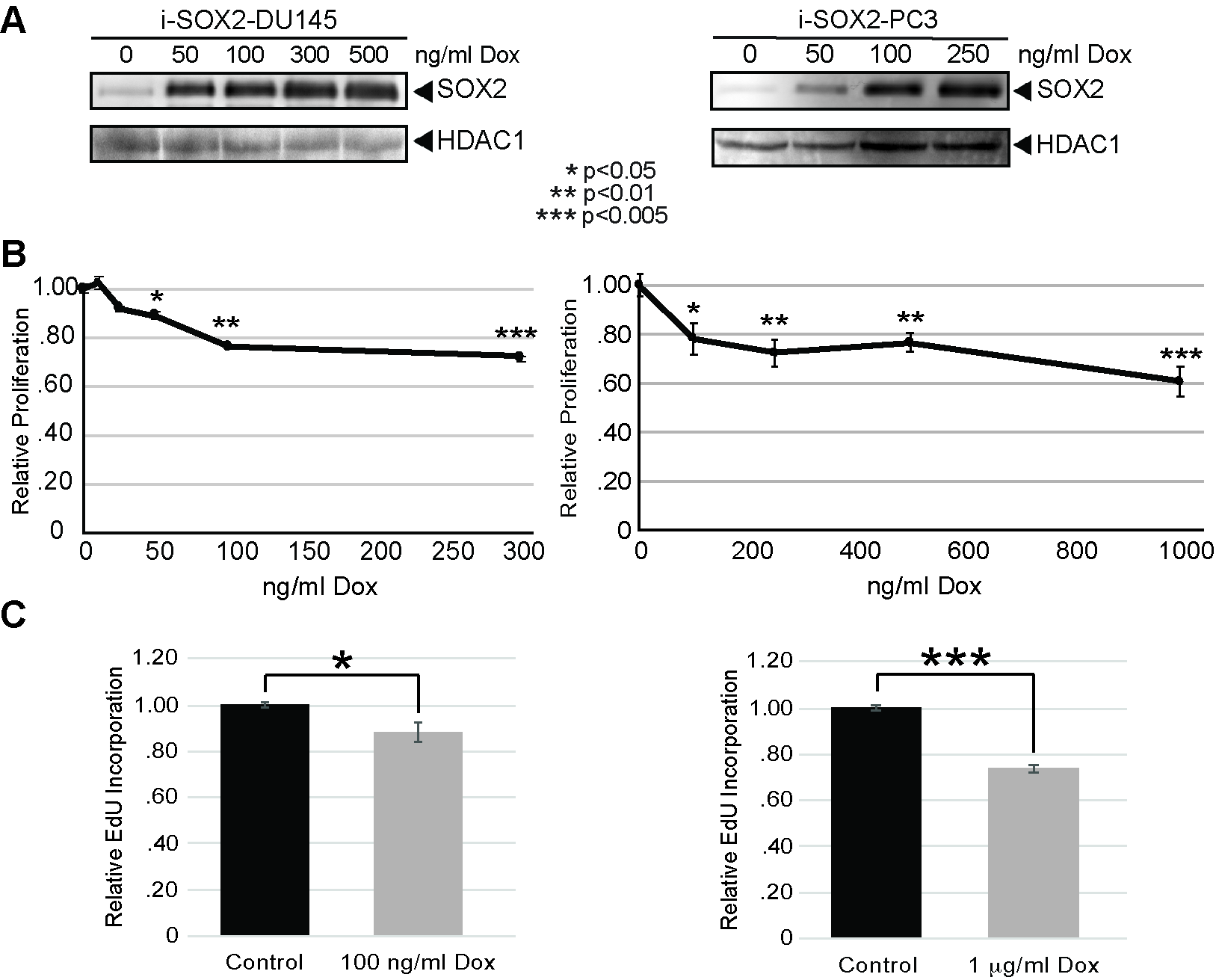

Supplement: Supplementary file 1 — Additional file 1: Figure S1. Elevating SOX2 inhibits the in vitro proliferation of i-SOX2-DU145 and i-SOX2-PC3 cells. A. Western blot analysis of SOX2 in whole cell extracts from i-SOX2-DU145 and i-SOX2-PC3 cells cultured for 48 h with Dox at the indicated doses. B. Cell proliferation of i-SOX2-DU145 and i-SOX2-PC3 cells were determined by MTT assay following 4 days culture in the presence or absence of Dox at the indicated doses. Error bars represent standard deviation. C. Relative EdU incorporation of i-SOX2-DU!45 and i-SOX2-PC3 cells following 4 days of culture in the presence or absence of Dox at the doses indicated. [file 12885_2020_7370_MOESM1_ESM.tif]

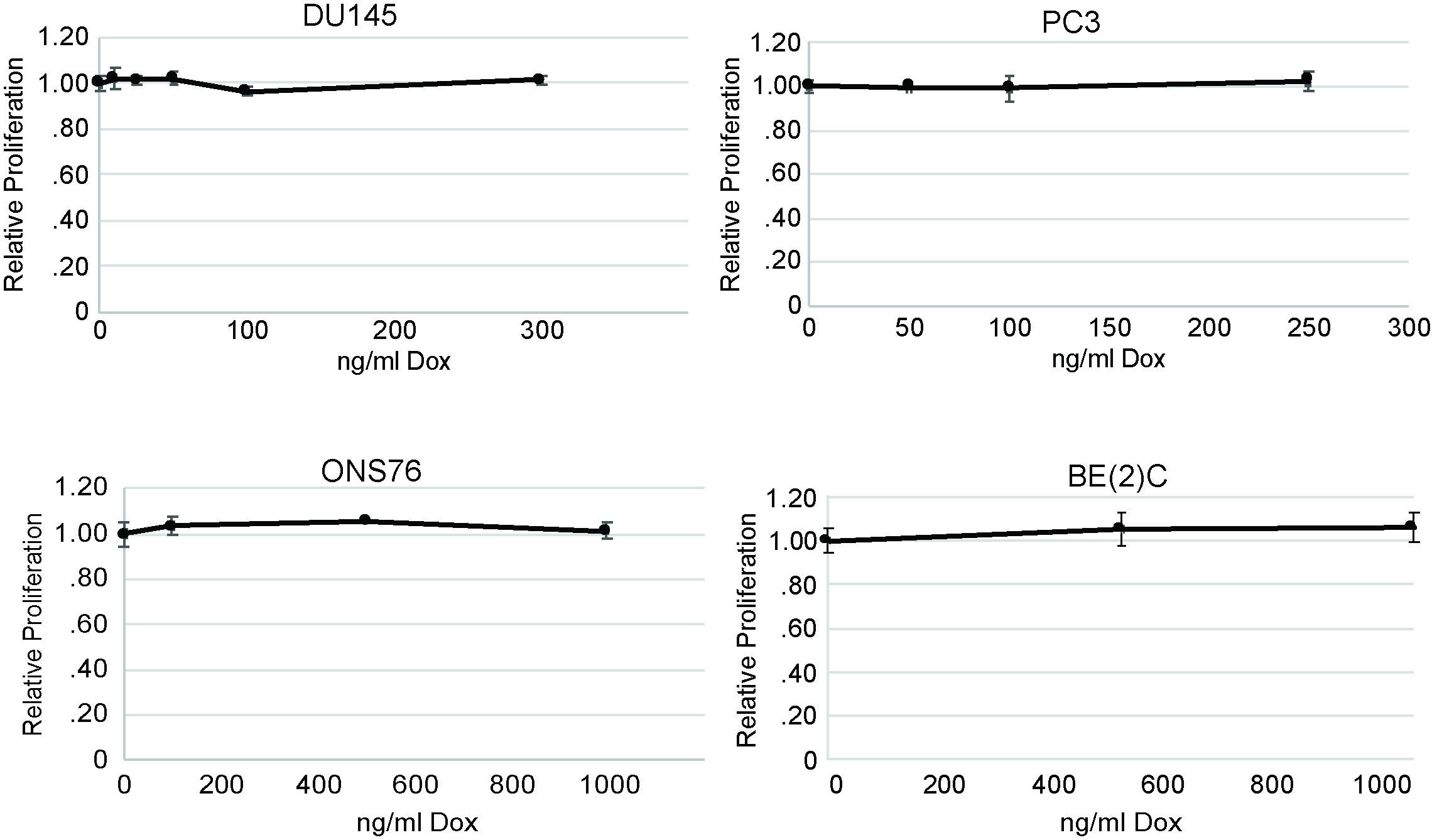

Supplement: Supplementary file 2 — Additional file 2: Figure S2. Dox treatment does not affect the proliferation of parental tumor cell lines. Proliferation of DU145, PC3, ONS76, and BE(2)C cells were determined by MTT assay following 4 days culture in the presence or absence of Dox at the indicated doses. Error bars represent standard deviation. [file 12885_2020_7370_MOESM2_ESM.tif]

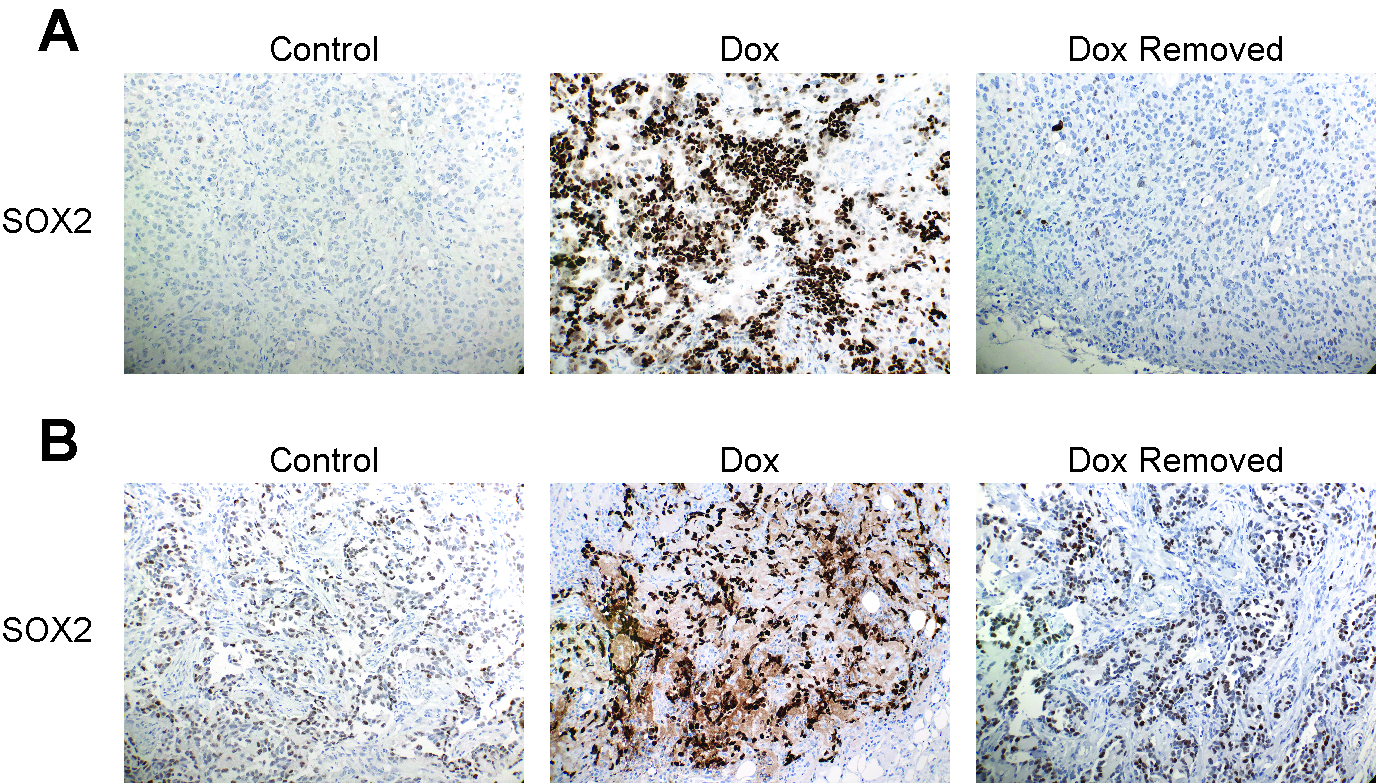

Supplement: Supplementary file 3 — Additional file 3: Figure S3. Dox treatment increases SOX2 expression in i-SOX2-DU145 and i-SOX2-ONS76 tumors in vivo. Immunohistochemical analysis of SOX2 expression in (A) i-SOX2-DU145 tumors and (B) i-SOX2-ONS76 tumors. [file 12885_2020_7370_MOESM3_ESM.tif]

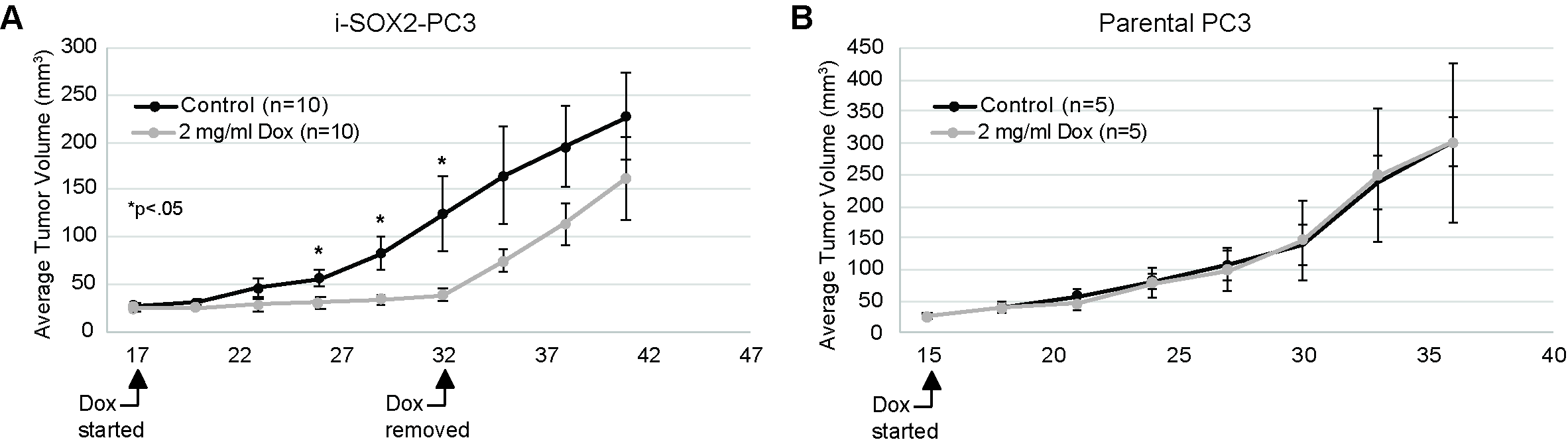

Supplement: Supplementary file 4 — Additional file 4: Figure S4. Elevating SOX2 in vivo reversibly inhibits the growth of i-SOX2-PC3 tumors. A. Subcutaneous i-SOX2-PC3 tumor growth of control and Dox-treated mice. Dox treatment was started and ended at the days indicated. Average tumor volumes are presented for control and Dox-treated groups. B. Subcutaneous parental PC3 tumor growth of control and Dox-treated mice. Dox treatment was started and stopped at the days indicated. Average tumor volumes are presented for control and Dox-treated groups. Error bars represent standard error of the mean; statistical significance was determined by two-tailed student’s t-test (*p<0.05). [file 12885_2020_7370_MOESM4_ESM.tif]

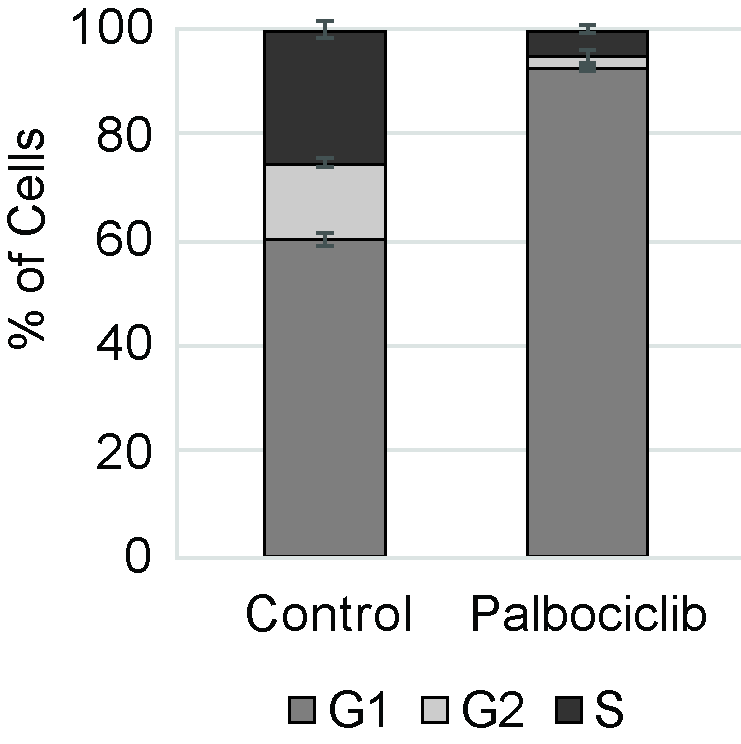

Supplement: Supplementary file 5 — Additional file 5: Figure S5. Palbociclib induces G1 cell cycle arrest. Cell cycle analysis was performed by flow cytometry after 4 days treatment with DMSO (control) and 1 μM Palbociclib. Error bars represent standard deviation. [file 12885_2020_7370_MOESM5_ESM.tif]

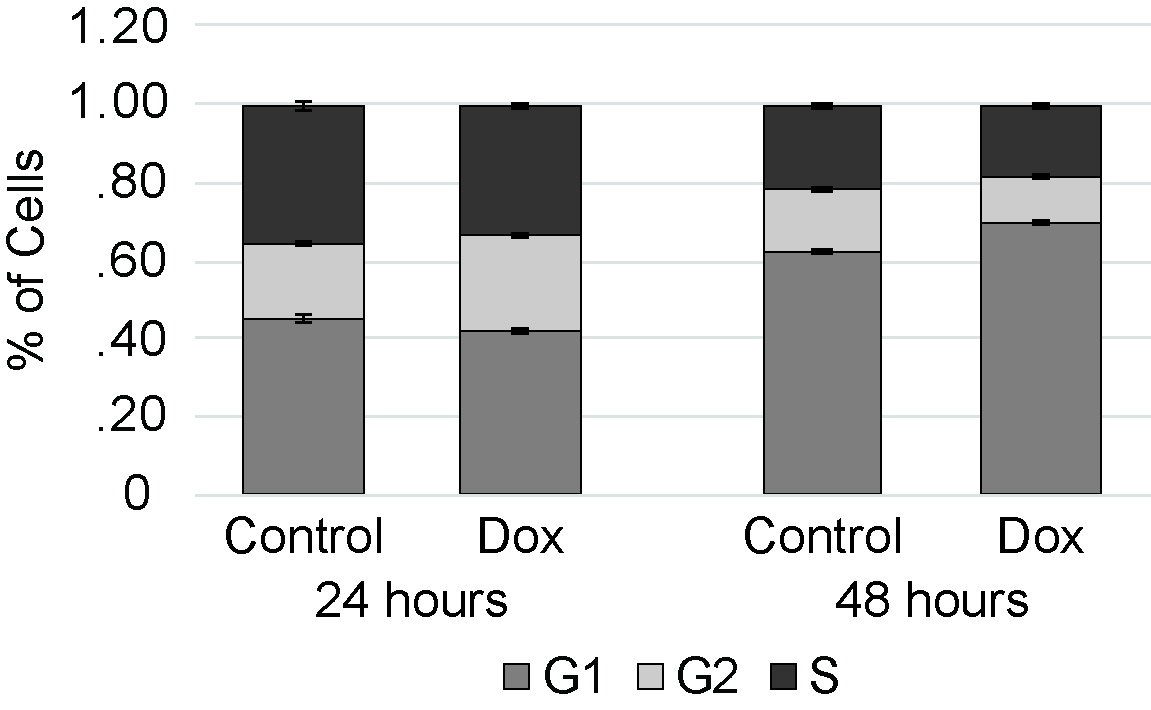

Supplement: Supplementary file 6 — Additional file 6: Figure S6. Elevating SOX2 does not dramatically alter the cell cycle distribution of i-SOX2-ONS76 cells at the one and two day time point. I-SOX2-ONS76 cells were subjected to cell cycle analysis after 24 or 48 h culture in the presence or absence of 100 ng/ml Dox. Error bars represent standard deviation. [file 12885_2020_7370_MOESM6_ESM.tif]

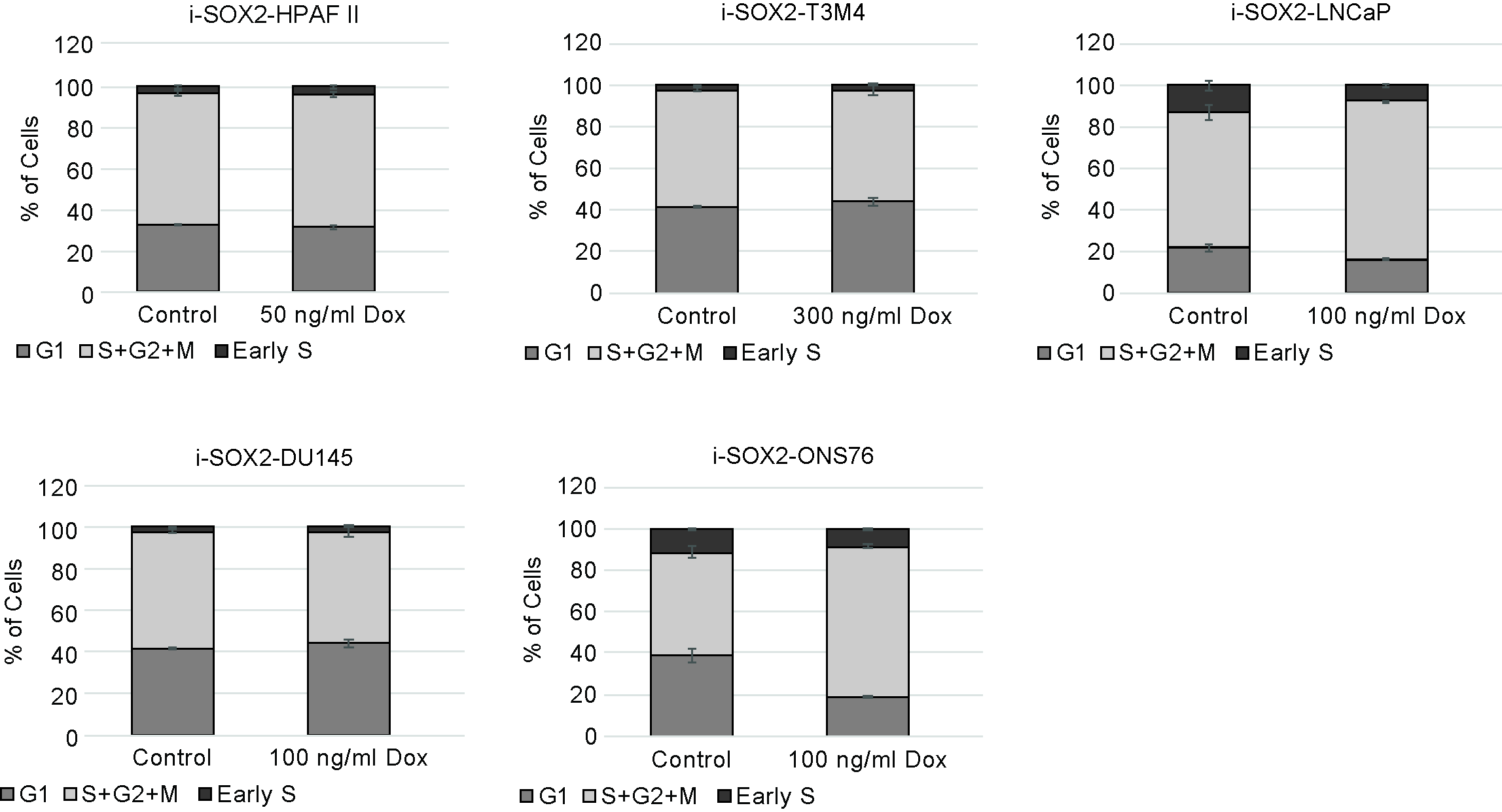

Supplement: Supplementary file 7 — Additional file 7: Figure S7. Elevating SOX2 does not significantly alter the cell cycle distribution of the majority of tumor cell lines examined. Cell cycle analysis was performed by flow cytometry using the FastFUCCI system after 4 days in the presence or absence of Dox at doses the indicated. [file 12885_2020_7370_MOESM7_ESM.tif]

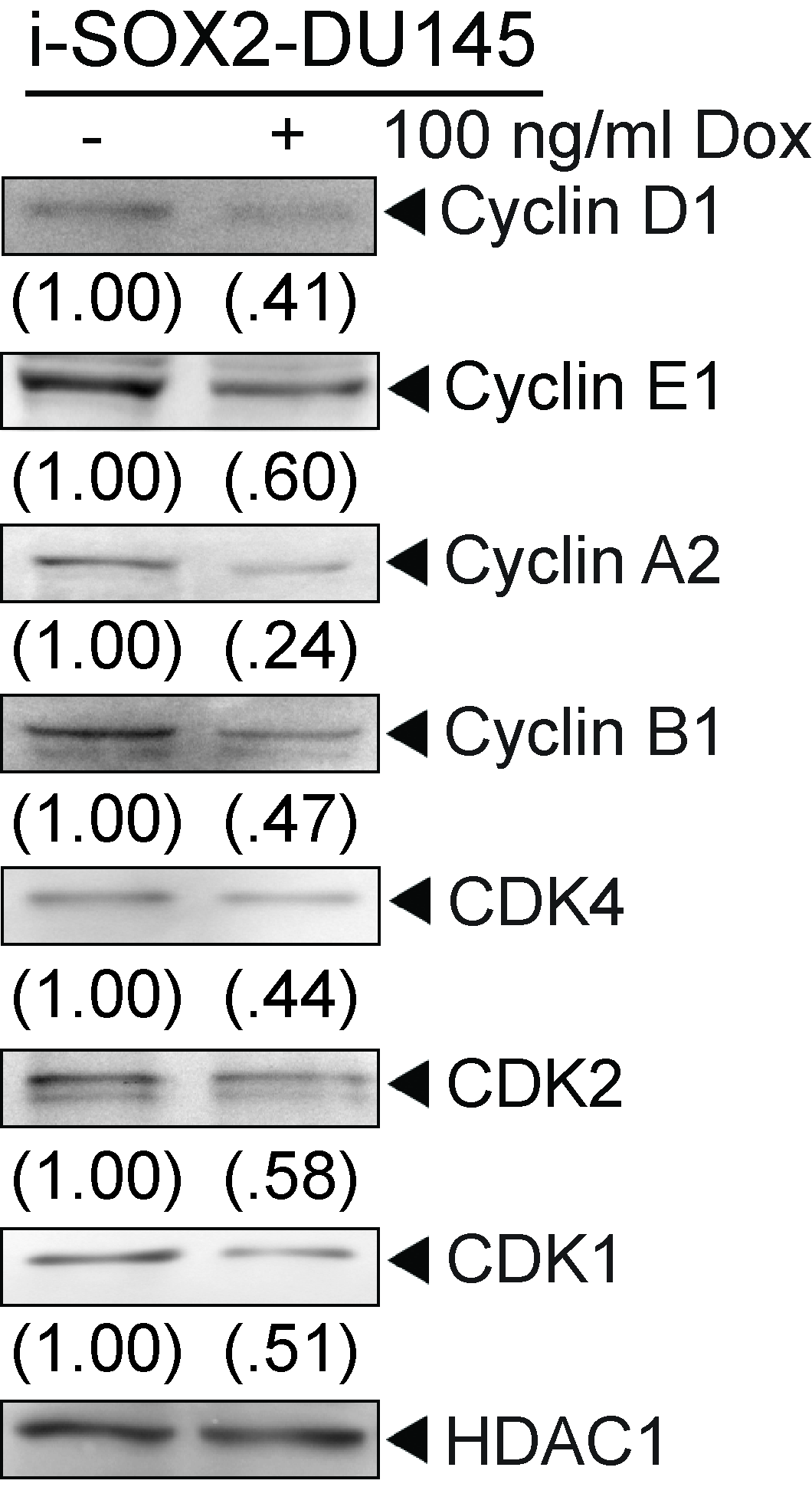

Supplement: Supplementary file 8 — Additional file 8: Figure S8. Elevating SOX2 in i-SOX2-DU145 cells decreases the expression of multiple cyclins and CDKs. Western blot analysis of i-SOX2-DU145 whole cell extracts harvested after 48 h growth in the presence or absence of 100 ng/ml Dox. [file 12885_2020_7370_MOESM8_ESM.tif]

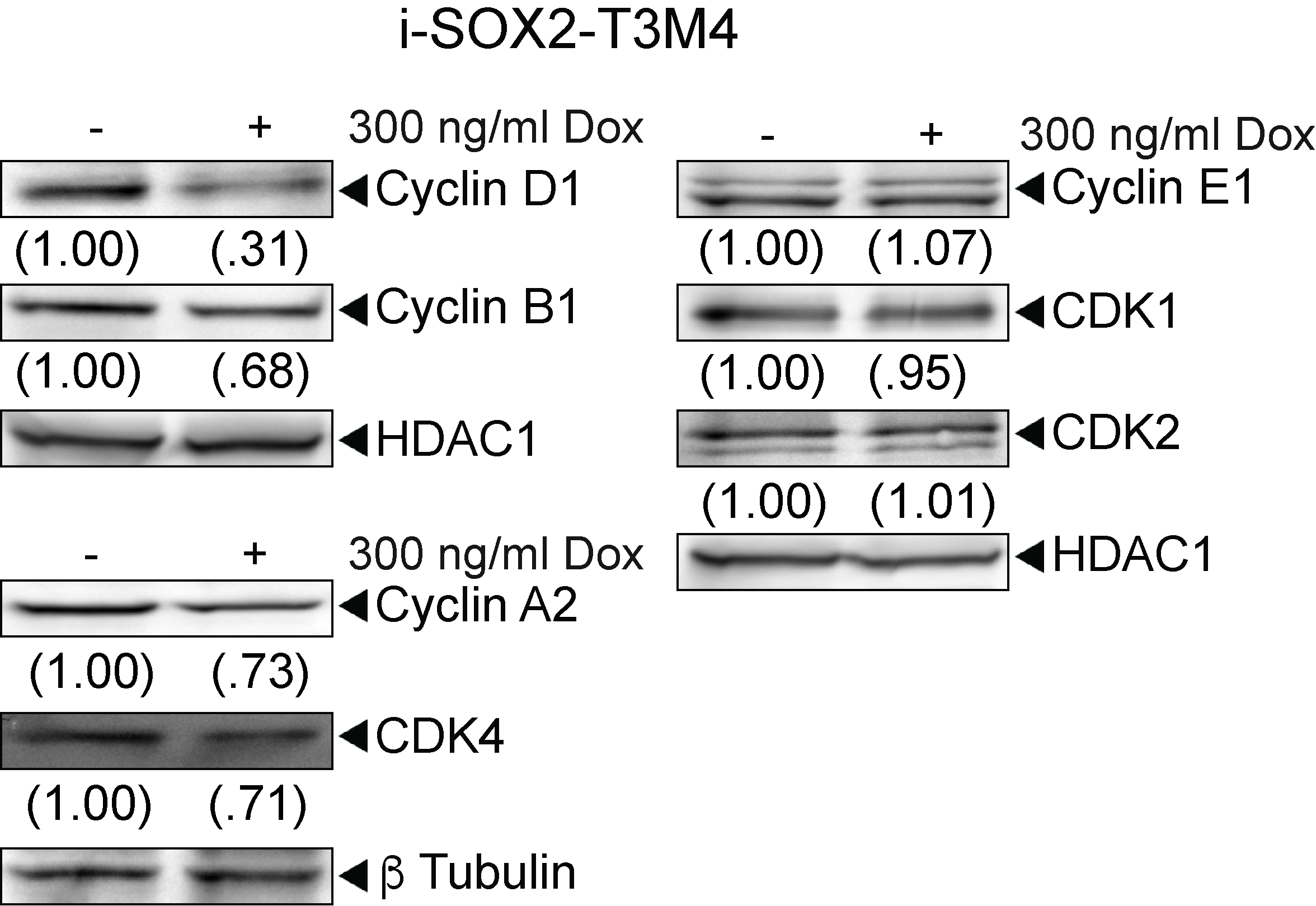

Supplement: Supplementary file 9 — Additional file 9: Figure S9. Elevating SOX2 in i-SOX2-T3M4 cells decreases the expression of cyclins and CDKs. Western blot analysis of i-SOX2-T3M4 whole cell extracts harvested after 48 h growth in the presence or absence of 300 ng/ml Dox. [file 12885_2020_7370_MOESM9_ESM.tif]

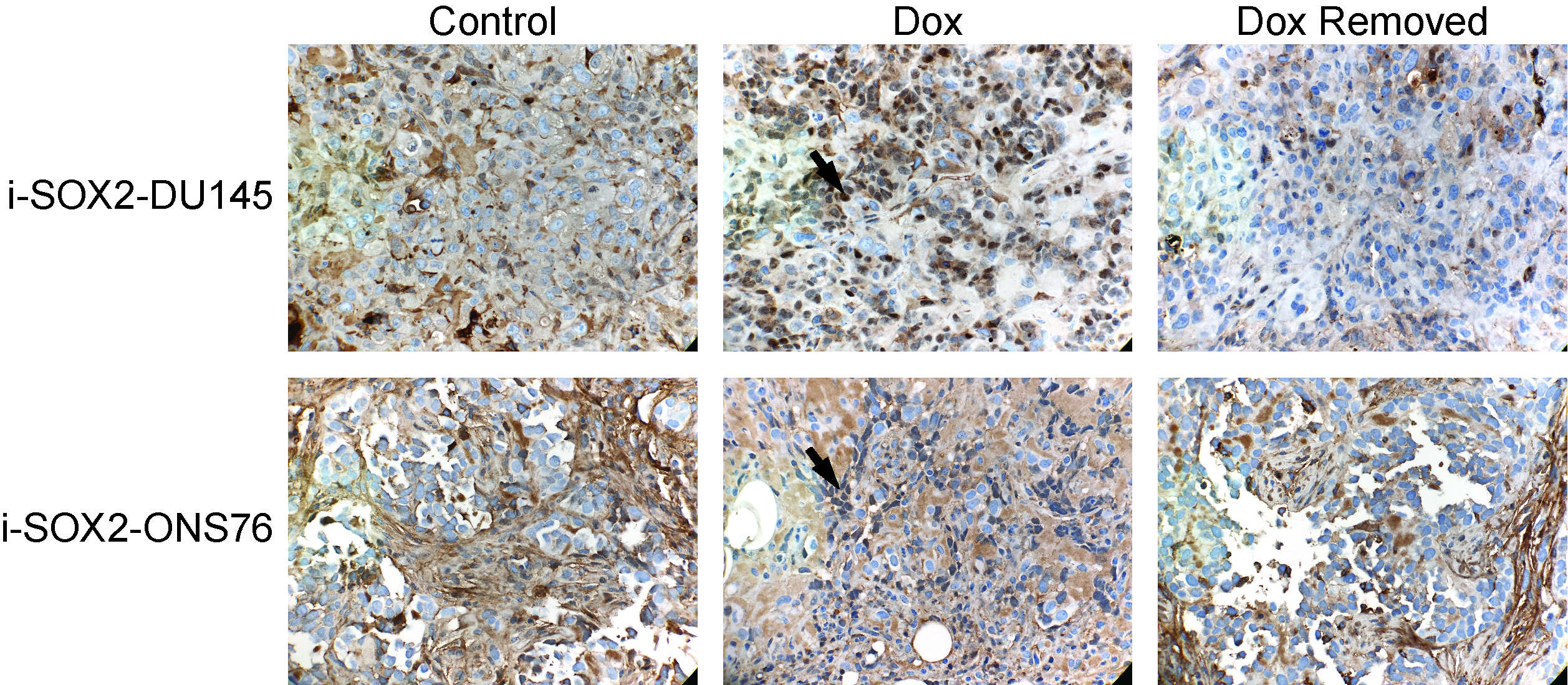

Supplement: Supplementary file 10 — Additional file 10: Figure S10. Elevating SOX2 in i-SOX2-DU145 and i-SOX2-ONS76 cells increases p27Kip1 nuclear localization. Immunohistochemical analysis of p27Kip1 expression in control, Dox-treated, and Dox-removed i-SOX2-DU145 and i-SOX2-ONS76 tumors. [file 12885_2020_7370_MOESM10_ESM.tif]

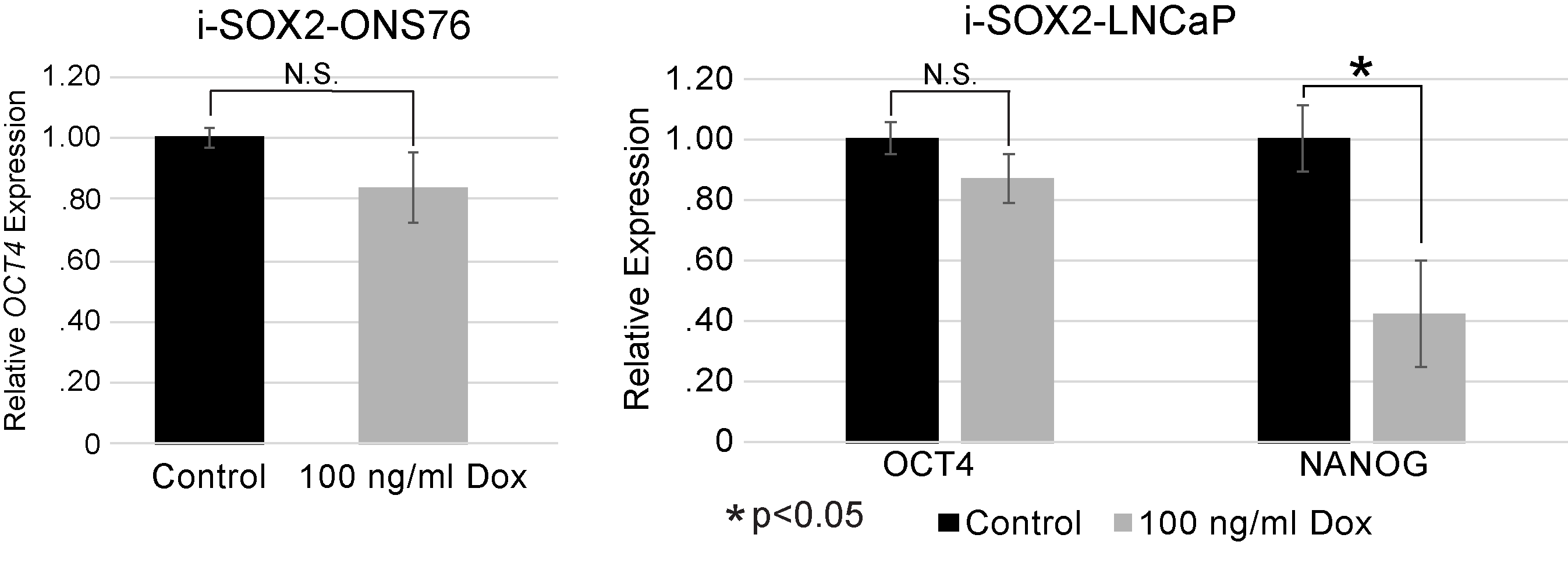

Supplement: Supplementary file 11 — Additional file 11: Figure S11. Elevating SOX2 does not alter the expression of OCT4 in i-SOX2-ONS76 or i-SOX2-LNCaP cells, but decreases NANOG expression in i-SOX2-LNCaP cells. RT-qPCR analysis of OCT4 and NANOG expression in mRNA from i-SOX2-ONS76 and i-SOX2-LNCaP cells were cultured in the presence or absence of 100 ng/ml Dox for 48 h. [file 12885_2020_7370_MOESM11_ESM.tif]

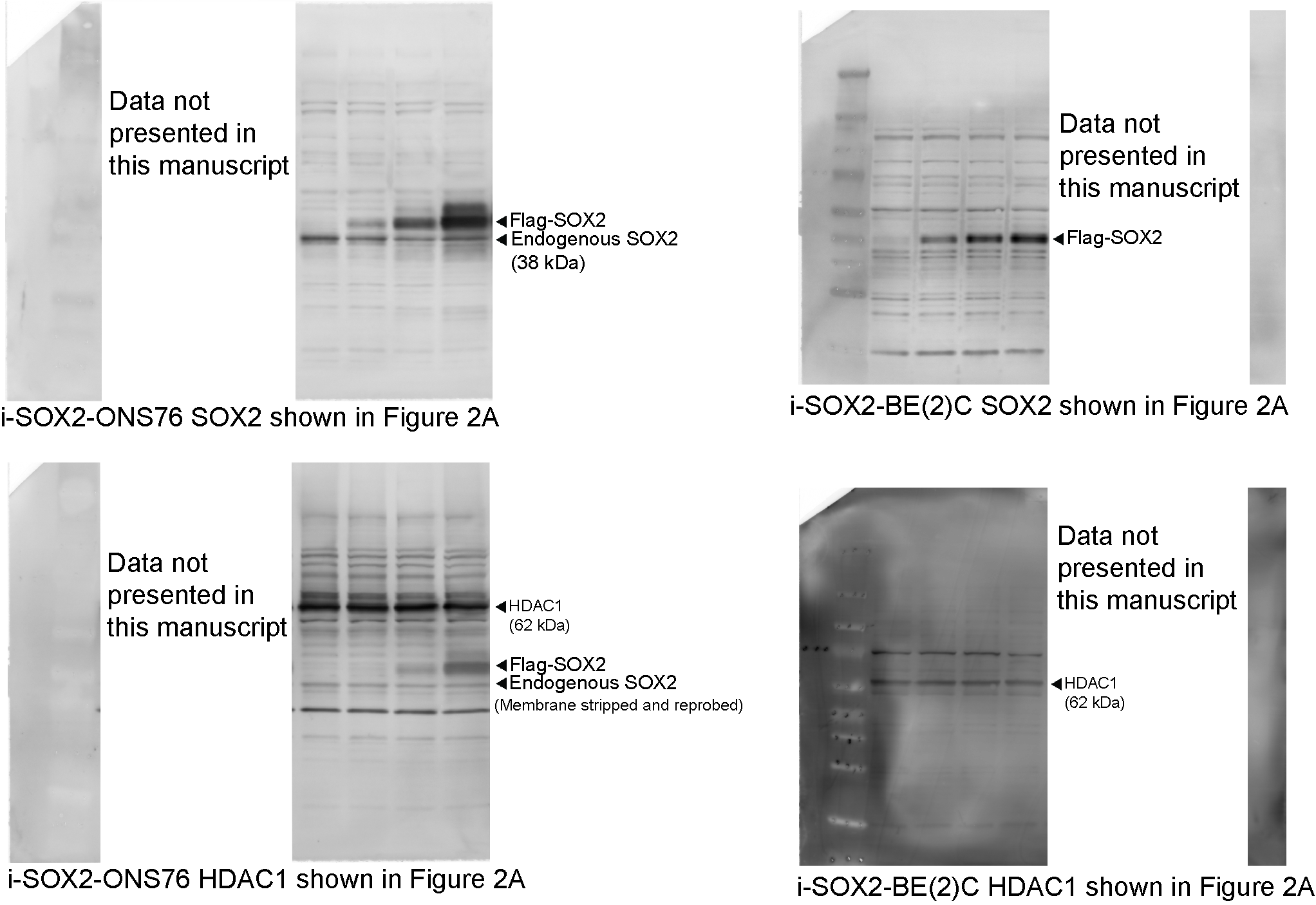

Supplement: Supplementary file 12 — Additional file 12: Figure 2A western blots. The original, full-length membrane images of western blot data in Figure 2A. [file 12885_2020_7370_MOESM12_ESM.tif]

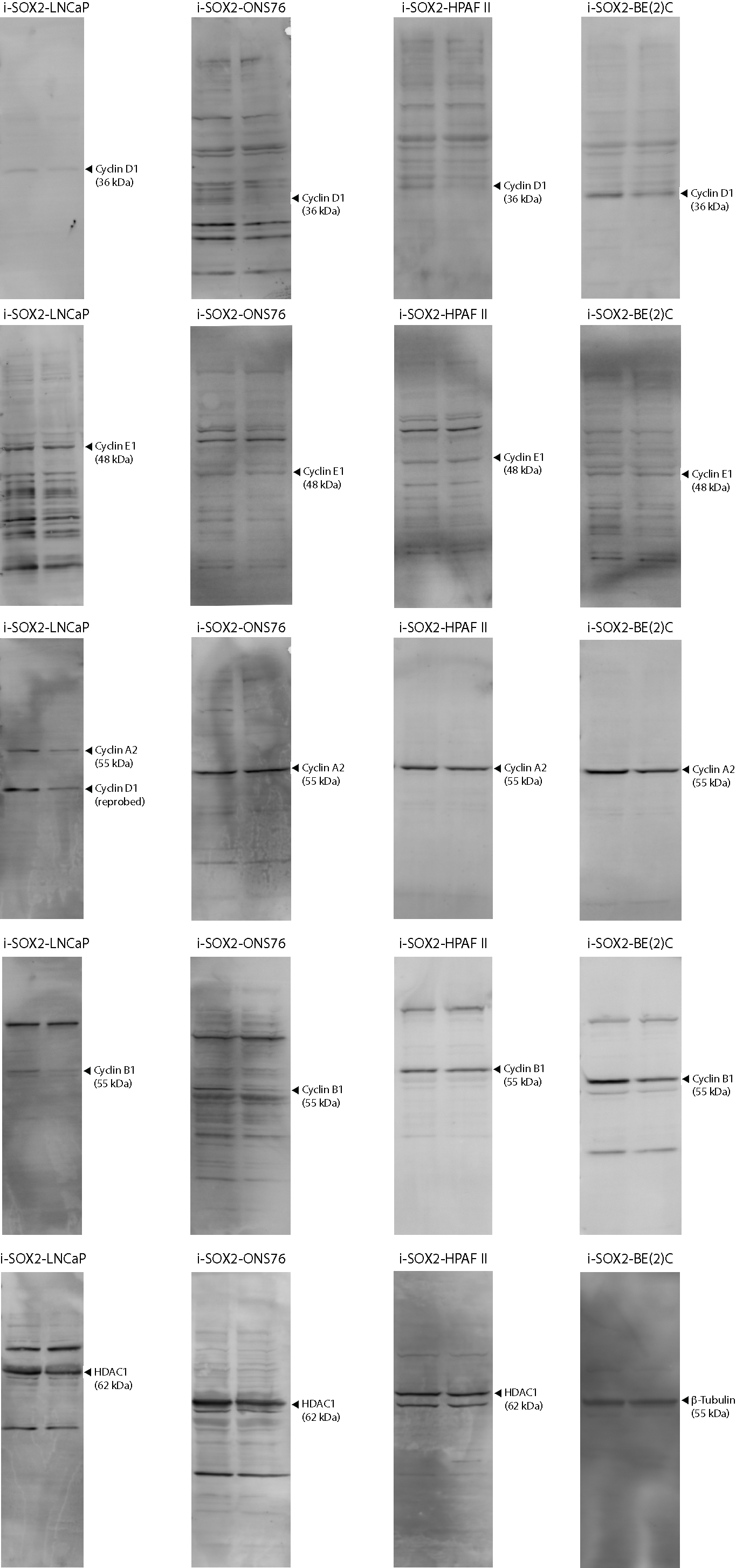

Supplement: Supplementary file 13 — Additional file 13: Figure 5A-D western blots. The original, full-length membrane images of western blot data in Figure 5A-D. Additional bands are due to repeated stripping and reprobing of the membrane. [file 12885_2020_7370_MOESM13_ESM.tif]

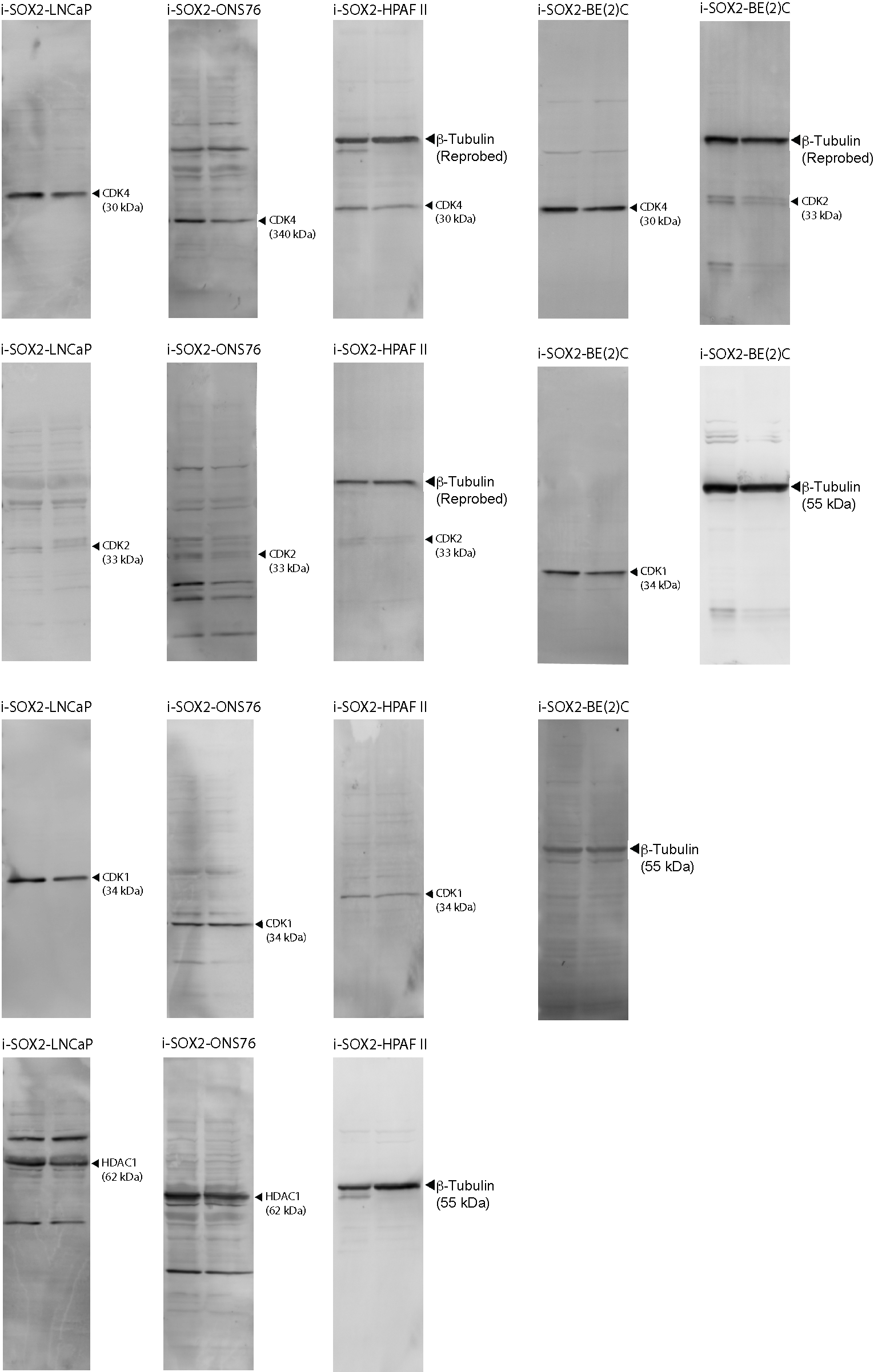

Supplement: Supplementary file 14 — Additional file 14: Figure 5E-H western blots. The original, full-length membrane images of western blot data in Figure 5E-H. Additional bands are due to repeated stripping and reprobing of the membrane. [file 12885_2020_7370_MOESM14_ESM.tif]

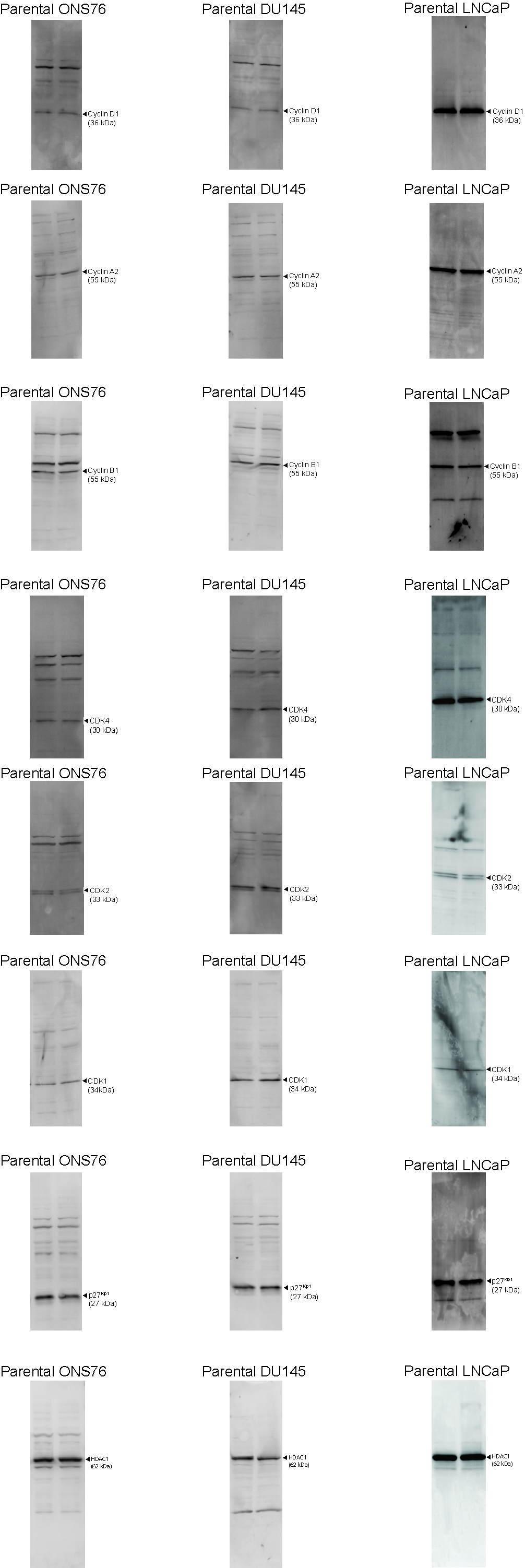

Supplement: Supplementary file 15 — Additional file 15: Figure 6 western blots. The original, full-length membrane images of western blot data in Figure 6. Additional bands are due to repeated stripping and reprobing of the membrane. [file 12885_2020_7370_MOESM15_ESM.tif]

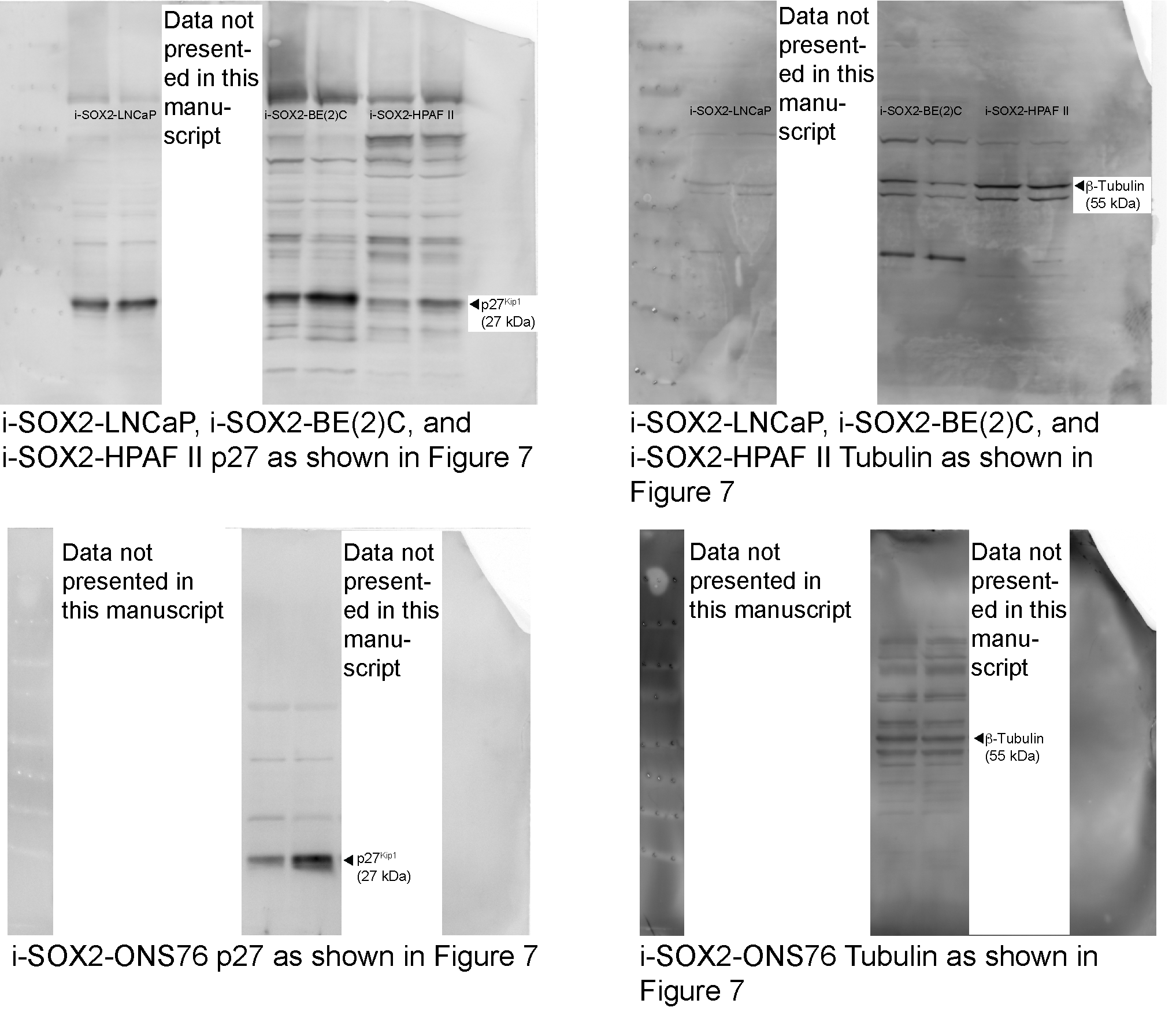

Supplement: Supplementary file 16 — Additional file 16: Figure 7 western blots. The original, full-length membrane images of western blot data in Figure 7. Additional bands are due to repeated stripping and reprobing of the membrane. [file 12885_2020_7370_MOESM16_ESM.tif]

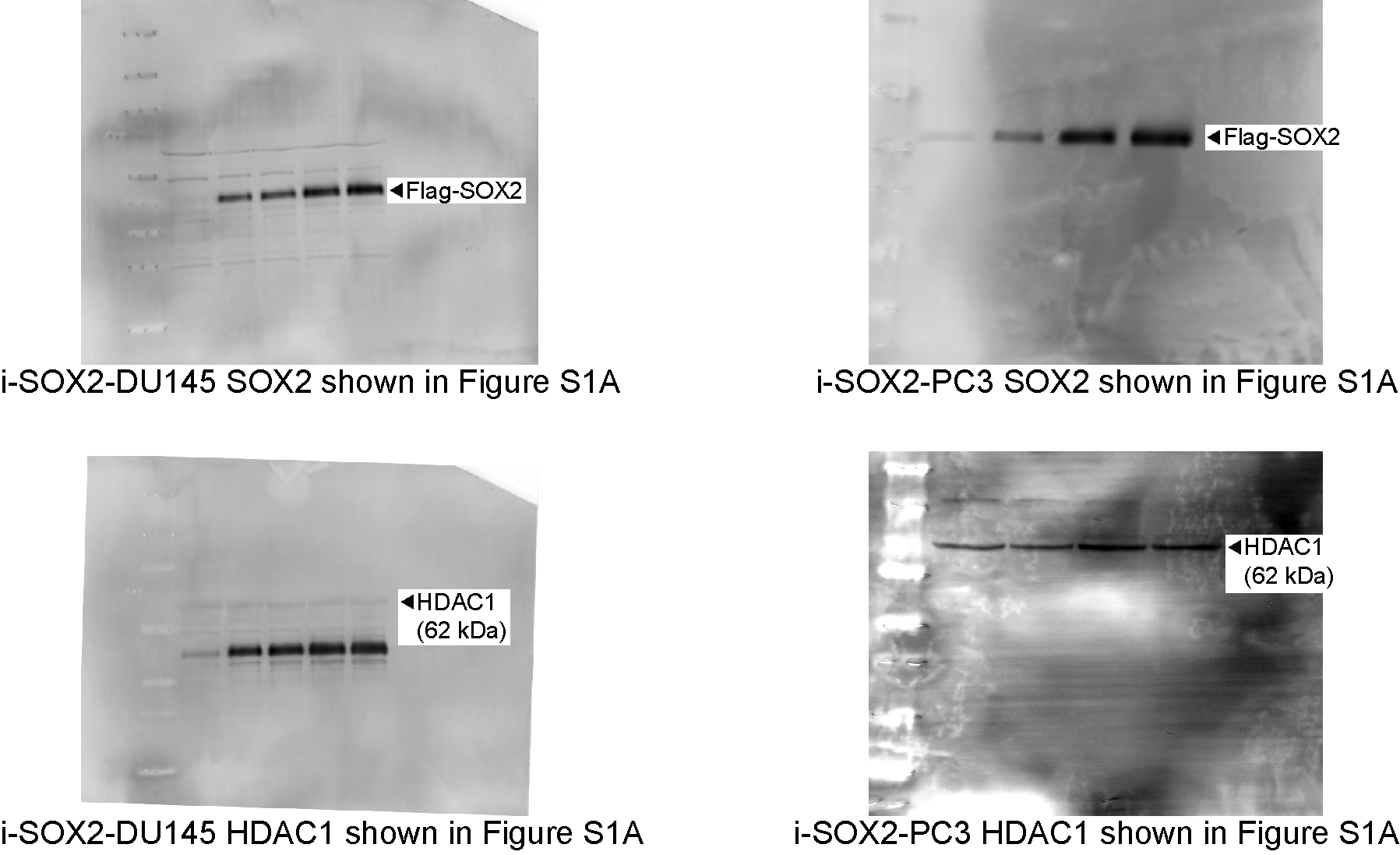

Supplement: Supplementary file 17 — Additional file 17: Figure S1A western blots. The original, full-length membrane images of western blot data in Figure S1A. [file 12885_2020_7370_MOESM17_ESM.tif]

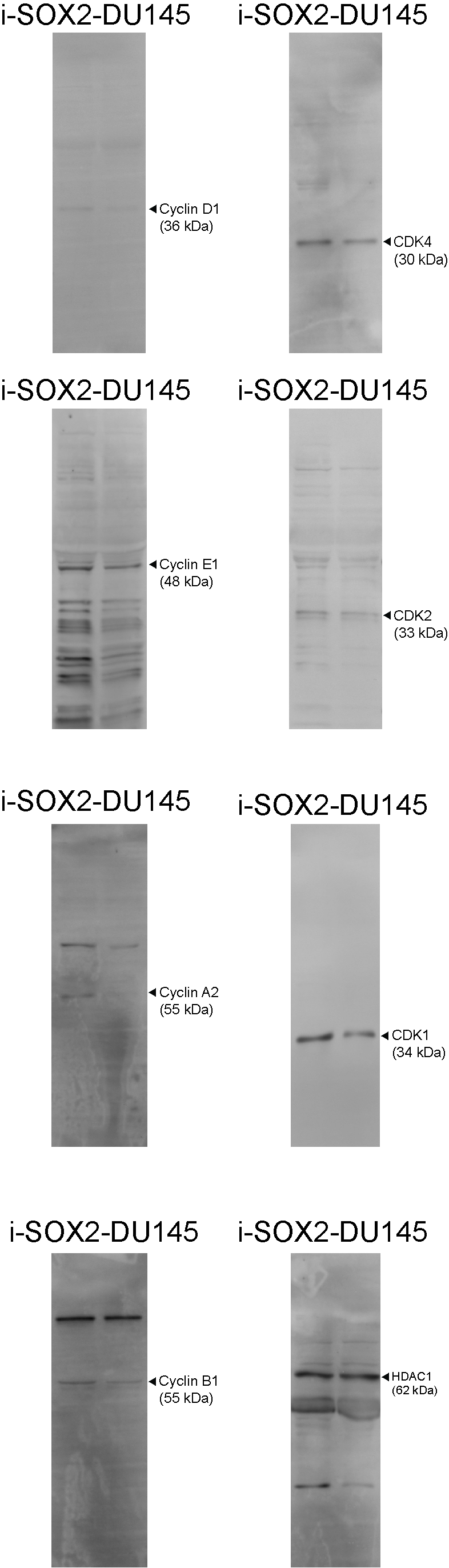

Supplement: Supplementary file 18 — Additional file 18: Figure S8 western blots. The original, full-length membrane images of western blot data in Figure S8. Additional bands are due to repeated stripping and reprobing of the membrane. [file 12885_2020_7370_MOESM18_ESM.tif]

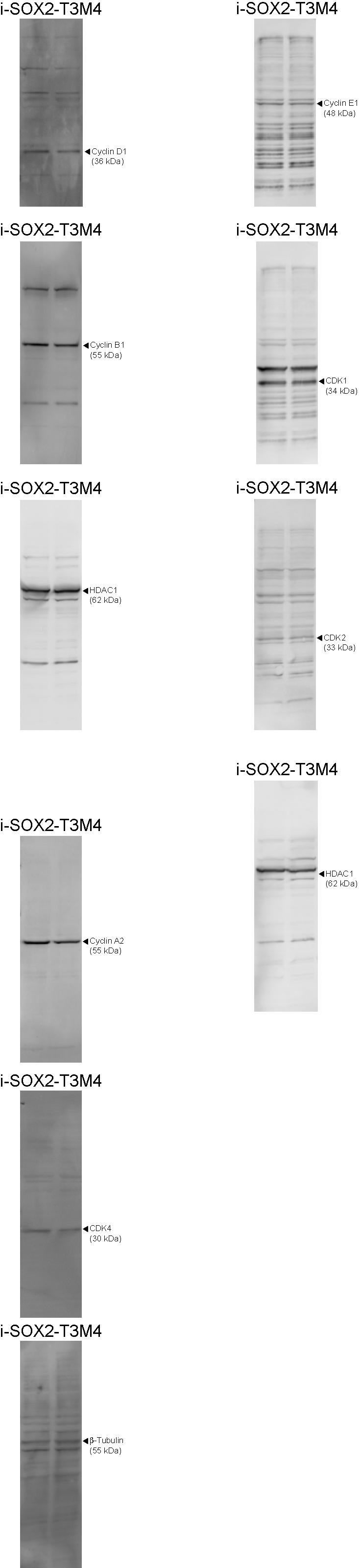

Supplement: Supplementary file 19 — Additional file 19: Figure S9 western blots. The original, full-length membrane images of western blot data in Figure S9. Additional bands are due to repeated stripping and reprobing of the membrane. [file 12885_2020_7370_MOESM19_ESM.tif]
